# Photosynthetic Responses of Spring Wheat Seedlings to Neutral, Alkaline, and Combined Salt Stresses

**DOI:** 10.3390/ijms27073060

**Published:** 2026-03-27

**Authors:** Yabo Dai, Jun Ye, Xuan Lei, Xiaobing Wang, Chenghao Zhang, Cundong Li, Zhanyuan Lu, Juan Li, Dejian Zhang

**Affiliations:** 1 Key Laboratory of Biology of Forage and Special Crops, Ministry of Education, School of Life Sciences, Inner Mongolia University, Hohhot 010021, China; dyb2869330798@163.com (Y.D.); yejun66@126.com (J.Y.); 15271197763@163.com (X.L.); m13210995641@163.com (C.Z.); 2 Inner Mongolia Academy of Agricultural & Animal Husbandry Sciences/Key Laboratory of Black Soil Conservation and Utilization, Ministry of Agriculture and Rural Affairs/Key Laboratory of Ecological Restoration and Pollution Control of Degraded Farmland of Inner Mongolia Autonomous Region, Hohhot 010031, China; xbwang1120@163.com (X.W.); lzhy281@163.com (Z.L.); 3 College of Agronomy, Hebei Agricultural University, Baoding 071001, China; nxylcd@hebau.edu.cn

**Keywords:** spring wheat, salt stress, genotype, structural equation, standardized regression coefficient, salt stress response slope

## Abstract

Soil salinization poses a severe threat to global wheat production, yet the physiological mechanisms underlying photosynthetic responses to neutral, alkaline, and combined salt stress remain poorly understood. This study systematically evaluated the photosynthetic physiology and salt tolerance of six spring wheat genotypes under three types of salt stress at varying concentrations. By integrating phenotypic data, gas exchange parameters, chlorophyll fluorescence indices, and biomass measurements, and applying structural equation modeling and multivariate analysis, key traits regulating biomass were identified. The results revealed significant interactions among salt stress type, genotype, and concentration on photosynthetic parameters. Structural equation modeling analysis revealed that under neutral salt stress, both gas exchange parameters and chlorophyll content had significant direct effects on seedling biomass, with standardized path coefficients of 0.421 and 0.400, respectively. Under alkaline and combined salt stresses, only chlorophyll content showed a significant direct effect on biomass, with standardized path coefficients of 0.873 and 0.790, respectively. Multiple regression analysis further identified key photosynthetic factors influencing growth under different stress types. Under neutral salt stress, phi (Ro) and E significantly affected biomass, whereas under alkaline and combined salt stresses, biomass was primarily co-regulated by phi (Ro) and phi (Eo). Based on a comprehensive evaluation of salt tolerance index, damage index, and biomass response, genotypes W06 and W02 exhibited the strongest overall salt tolerance. This study systematically elucidates the differential response mechanisms of photosynthesis in spring wheat under distinct salt stress types, providing an important theoretical basis and elite germplasm resources for breeding salt-tolerant wheat varieties.

## 1. Introduction

Wheat (*Triticum aestivum* L.) is one of the world’s most important food crops, providing approximately 20% of human caloric and protein intake. It is cultivated on over 219 million hectares globally, with annual production exceeding 760 million tons [1,2,3]. In China, wheat is grown on approximately 24 million hectares, yielding about 134 million tons annually. Spring wheat accounts for about 1.24 million hectares and 5.83 million tons, mainly in arid and semi-arid regions such as Inner Mongolia, Xinjiang, and Gansu [4,5,6]. Due to its short growth cycle, spring wheat plays an important role in national food security and in optimizing cropping systems [4,5,6].

Soil salinization is a growing threat to global agriculture. Approximately 424 million hectares of topsoil and a comparable area of subsoil are currently affected by salinity, primarily due to low rainfall, high evaporation, and improper irrigation practices. It is projected that by 2050, over 50% of arable land may be salinized [7,8]. In China, saline-alkaline soils cover about 36.7 million hectares, accounting for nearly 4.88% of the country’s arable land [7,8]. Salt stress impairs wheat growth by inducing osmotic stress, ion toxicity, oxidative damage, and increased pH, thereby disrupting metabolism, inhibiting development, and reducing yield.

The seedling stage is particularly sensitive to salt stress, as it is critical for organ formation and photosynthetic establishment. Salt stress at this stage inhibits root development, accelerates leaf senescence, and damages the photosynthetic apparatus [9,10]. Mechanistically, salt stress limits photosynthesis through stomatal closure induced by osmotic stress, which reduces CO_2_ uptake, and through non-stomatal limitations, such as reactive oxygen species (ROS)-induced damage to photosystem II (PSII), disruption of thylakoid membranes, and impaired electron transport [11,12]. These changes result in declines in chlorophyll content, PSII efficiency (Fv/Fm), electron transport rate (ETR), and overall carbon assimilation [13].

Given the complexity of salt stress responses, systematic analyses integrating multiple physiological traits are needed to identify key determinants of salt tolerance. This study aims to (1) characterize the differential photosynthetic responses of spring wheat seedlings to neutral, alkaline, and combined salt stress; (2) identify key traits influencing biomass accumulation; and (3) evaluate and screen salt-tolerant germplasm resources during the seedling stage. Six spring wheat genotypes were assessed under varying salt types and concentrations using chlorophyll content, gas exchange, and chlorophyll fluorescence parameters. The findings provide a theoretical basis and germplasm resources for breeding salt-tolerant wheat varieties.

## 2. Results

### 2.1. Three-Way ANOVA of Wheat Seedlings Under Different Salt Stress Types

Table 1: A three-way ANOVA was conducted to systematically evaluate the effects of salt type (ST), genotype (G), and salt concentration (SC) on seedling physiological traits. In the single-factor effect analysis, ST significantly influenced all traits except gas exchange parameter Ci (*p* = 0.715) and chlorophyll fluorescence parameters ETo/RC (*p* = 0.762) and REo/RC (*p* = 0.053). G significantly influenced most physiological traits except RCR (*p* = 0.081), Ci (*p* = 0.748), and ABS/RC (*p* = 0.090). Furthermore, the effect of SC also had no significant impact on Ci (*p* = 0.695), mirroring the effect of salt type.

Regarding two-way interactions, the ST × G interaction significantly influenced most parameters, except for seedling DW (*p* = 0.593), RCR (*p* = 0.927), and gas exchange parameters A (*p* = 0.815) and Ci (*p* = 0.887), which did not reach significance. The ST × SC interaction had a greater effect, with no significant influence on seedling DW (*p* = 0.779), gas exchange parameters A (*p* = 0.793) and Ci (*p* = 0.877), as well as the chlorophyll fluorescence parameter REo/RC (*p* = 0.079). The G × SC interaction showed no significant effects on seedling DW (*p* = 0.963), RCR (*p* = 0.789), gas exchange parameters A (*p* = 0.778) and Ci (*p* = 0.999), or chlorophyll SPAD (*p* = 0.268).

The three-way interaction (ST × G × SC) significantly affected only specific gas exchange parameters (E, GSW) and chlorophyll fluorescence parameters (Fv/Fm, Sm, ABS/RC, DIo/RC, ETo/RC, REo/RC, psi (Eo), phi (Eo), delta (Ro), phi (Ro), ETo/CSm, REo/CSm, PIabs).

### 2.2. Distribution of Salt Tolerance Index (STI) and Salt Damage Index (SDI) for Each Trait Under Different Salt Stress Types

**Figure 1:**
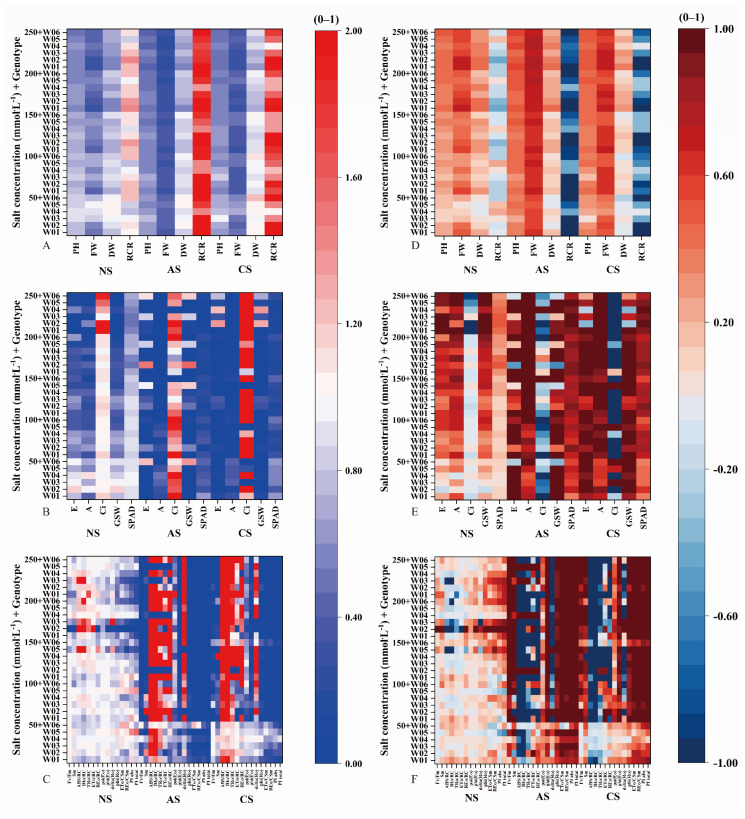
Heatmaps of the salt tolerance index (STI) and salt damage index (SDI) for physiological traits under (**A**,**D**) neutral, (**B**,**E**) alkaline, and (**C**,**F**) combined salt conditions stress.

Under neutral salt stress (Figure 1A, Table S1), STI showed significant variation among genotypes. The heatmap showed a gradient of tolerance, with some genotypes, such as W05 and W06, consistently showing high STI across different salt concentrations. Under alkaline salt stress (Figure 1B, Table S1), the STI pattern differed from that under neutral conditions. Genotypes including W03 and W04 demonstrated relatively higher STI, suggesting specific adaptation to alkaline environments. In the case of combined salt stress (Figure 1C, Table S1), STI values were generally lower across all genotypes compared to other stress types; however, genotypes such as W02 and W06 maintained relatively higher STI, indicating some resilience under combined stress.

Regarding SDI under neutral salt stress (Figure 1D, Table S1), genotypes like W01 and W02 showed lower values, indicating less damage and potential adaptation to neutral salinity. In contrast, genotypes including W05 and W06 exhibited higher SDI, suggesting increased susceptibility. Under alkaline salt stress (Figure 1E, Table S1), SDI was generally higher across most genotypes, with W03 and W04 showing the least damage, indicating inherent alkaline resistance. Under combined salt stress (Figure 1F, Table S1), SDI reached the highest levels among all stress conditions, indicating severe overall impairment, although genotypes such as W02 and W06 showed relatively lower SDI values.

### 2.3. Correlation Network Analysis Among Traits Under Different Treatments

**Figure 2:**
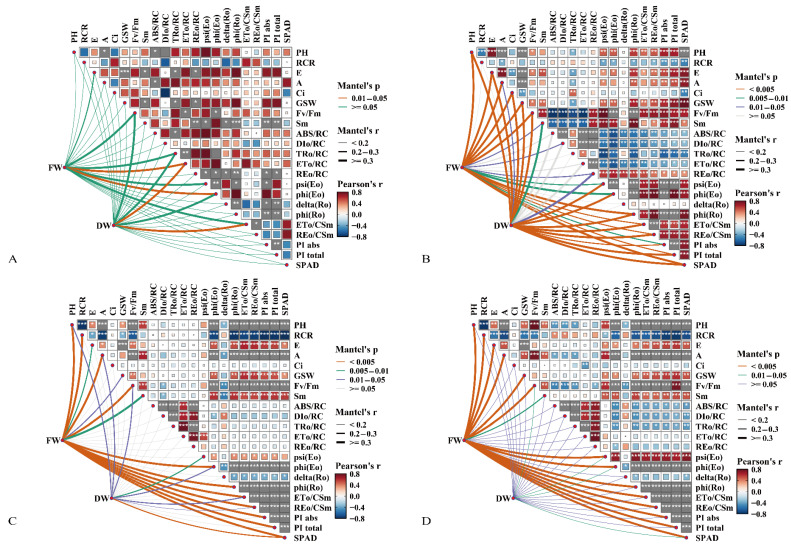
Correlation network analysis of physiological traits shown by heatmap visualization across different treatment regimes: control (**A**), neutral salt stress (**B**), alkaline salt stress (**C**), and combined salt stress (**D**), * *p* < 0.05, ** *p* < 0.01, *** *p* < 0.001.

Network correlation analysis revealed different patterns of association with biomass (FW, DW) across treatments (Figure 2). Under control conditions, ETo/RC showed significant correlations with both FW and DW. Additionally, FW correlated with phi (Eo). In addition, DW was associated with ETo/CSm (Figure 2A). Under neutral salt stress, FW and DW showed significant correlations with phenotypic traits (PH), gas exchange parameters (E, A, Ci, GSW), chlorophyll fluorescence parameters (Fv/Fm, Sm, REo/RC, psi (Eo), phi (Eo), phi (Ro), ETo/CSm, REo/CSm, PIabs, PItotal), and chlorophyll content (SPAD) (Figure 2B). Under alkaline salt stress, significant FW/DW correlations persisted with phenotypic traits (PH), gas exchange parameters (A), chlorophyll fluorescence parameters (Fv/Fm, phi (Eo), ETo/CSm, REo/CSm), and chlorophyll content (SPAD) (Figure 2C). Combined salt stress further refined these associations, with both biomass parameters specifically correlating with chlorophyll fluorescence parameters (Fv/Fm, psi (Eo), phi (Eo)) and chlorophyll content (SPAD) (Figure 2D).

### 2.4. Effects of Different Types of Salt Stress Levels on Seedling Biomass

**Figure 3:**
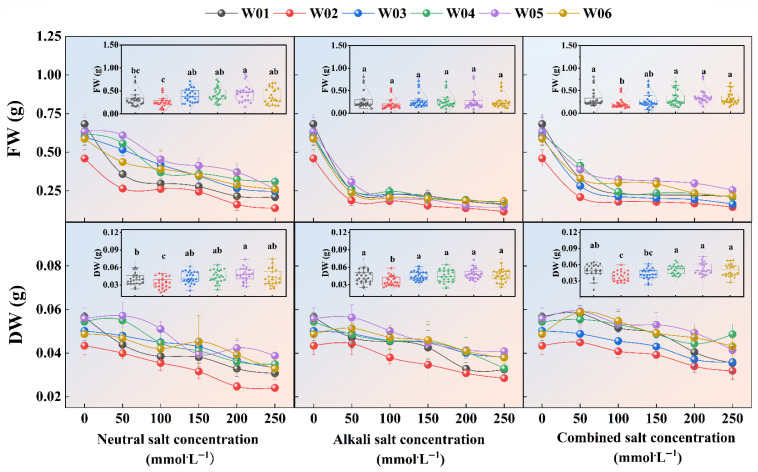
Characteristics of the cumulative distribution of seedling biomass under different salt types (neutral salt, alkaline salt, and combined salt) stress, and how it changes with increasing concentration, different letters (a, b, c) indicate significant differences at *p* < 0.05 (Duncan’s multiple range test), while the same letter indicates no significant difference. The letters are assigned in descending order of the mean values.

As shown in Figure 3, fresh weight (FW) and dry weight (DW) decreased in a concentration-dependent manner for all genotypes under all salt-stress conditions. The FW reduction between 0–50 mmol·L^−1^ was less pronounced under neutral salt stress than under alkaline or combined stresses, with FW consistently showing steeper declines than DW across stress types. Significant genotypic differences in FW and DW distribution were observed under neutral salt stress, particularly distinguishing genotypes W02 and W05. Alkaline stress caused significant DW variation only in W02, while combined stress reflected neutral stress patterns: FW showed notable differences between W02 and W01/W04/W05/W06, and DW between W02 and W04/W05/W06.

Table A1 further shows significant genotypic differences in FW at all concentrations, except 100 mmol·L^−1^, under neutral stress. Alkaline stress caused significant FW variations, specifically at 0, 50, and 250 mmol·L^−1^. Under combined stress, all concentrations exhibited substantial differences in FW. Analysis of DW in Table A2 showed significant genotypic variation at 50, 100, and 200 mmol·L^−1^ under neutral stress, and only at 250 mmol·L^−1^ under alkaline stress. In contrast, no significant differences in DW were observed under combined stress conditions.

### 2.5. Structural Equation Modeling of Biomass Contributions from Photosynthetic Parameters Under Contrasting Salt-Stress Regimes

**Figure 4:**
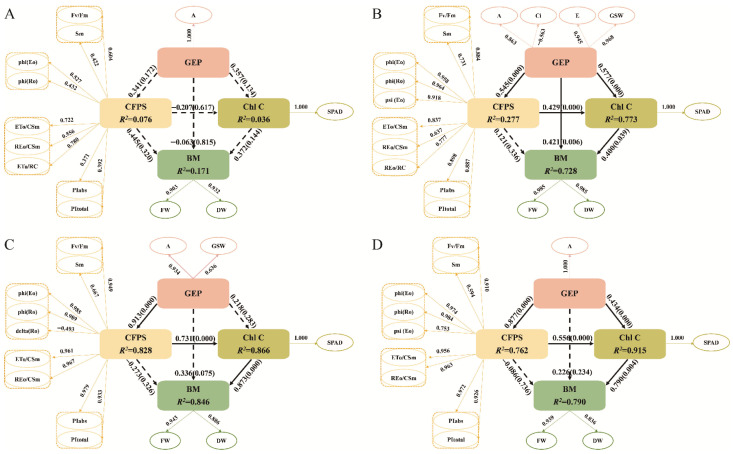
Structural equation modeling of photosynthetic contributions to biomass under salt-stress conditions: (**A**) control, (**B**) neutral salt, (**C**) alkaline salt, (**D**) combined salt. Path significance: solid arrow (*p* ≤ 0.05), dashed arrow (*p* > 0.05). Abbreviations: GEP (gas exchange parameters), CFPS (chlorophyll fluorescence parameters), Chl C (chlorophyll content), BM (biomass), Chlorophyll fluorescence parameters are functionally classified into four categories: PSII primary photochemical reaction and energy capture parameters, light energy conversion efficiency parameters, electron transport flux per reaction center parameters, and performance index parameters.

**Figure A1:**
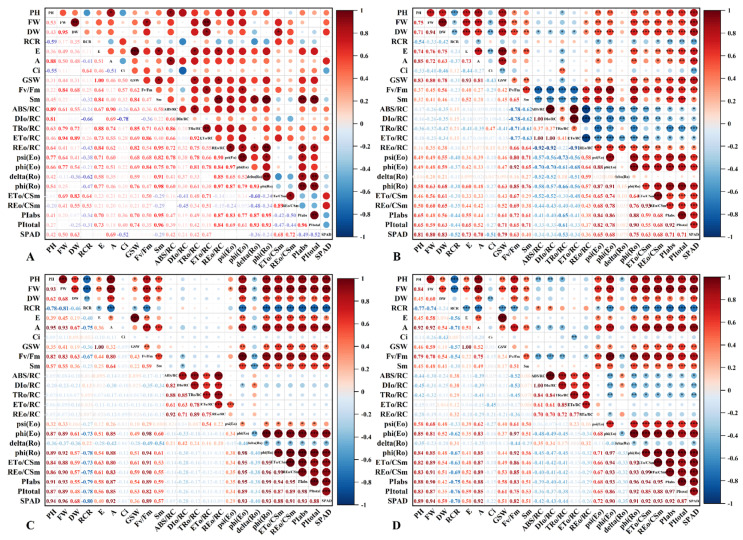
Correlation matrix of physiological traits across different experimental treatments: control (**A**), neutral salt stress (**B**), alkaline salt stress (**C**), and combined salt stress (**D**), * *p* < 0.05, ** *p* < 0.01, *** *p* < 0.001.

Key traits linked to FW and DW were identified using Pearson correlation heatmaps (Figure A1) and network analyses (Figure 2), which were then used as inputs for partial least squares-based structural equation modeling (PLS-SEM). Under control conditions, ETo/RC was the only trait showing significant correlations with both FW and DW, leading to the construction of PLS-SEM using this intersectional trait. This model showed that gas exchange parameters (GEP) had minimal direct impact on biomass (BM) (*β* = −0.063), but significant indirect effects through chlorophyll fluorescence parameters (CFPS) and chlorophyll content (Chl C) (*β* = 0.258). CFPS had notable direct effects (*β* = 0.445; total effect *β* = 0.368), and Chl C influenced BM only through direct pathways (*β* = 0.372) (Figure 4A, Table 2).

Under neutral salt stress (Figure 4B, Table S2), GEP had significant direct effects on BM (*β* = 0.421, *p* < 0.05), with indirect effects through CFPS and Chl C also contributing significantly (*β* = 0.390). Chl C maintained its direct effects on BM (*β* = 0.400, *p* = 0.039). CFPS influences both directly (*β* = 0.121) and via Chl C (*β* = 0.171) (Table 2).

Alkaline salt stress conditions (Figure 4C, Table 2, Table S2) altered these relationships: GEP maintained direct effects (*β* = 0.336) while increasing indirect effects (*β* = 0.525), CFPS showed a negative direct influence (*β* = −0.273) but a positive total effect (*β* = 0.366), and Chl C displayed strong direct effects (*β* = 0.873, *p* = 0.000).

Under combined salt stress (Figure 4D, Table 2, Table S2), GEP’s direct effect decreased (*β* = 0.226), while indirect effects increased (*β* = 0.631). CFPS showed little direct impact (*β* = −0.086) but significant Chl C-mediated indirect effects (*β* = 0.426), collectively leading to Chl C’s strong overall effect on BM (*β* = 0.766).

### 2.6. Linear Fitting of the Growth of Wheat Genotypes Under Different Types of Salt Stress and Distribution of Their Salt Tolerance Thresholds

**Figure 5:**
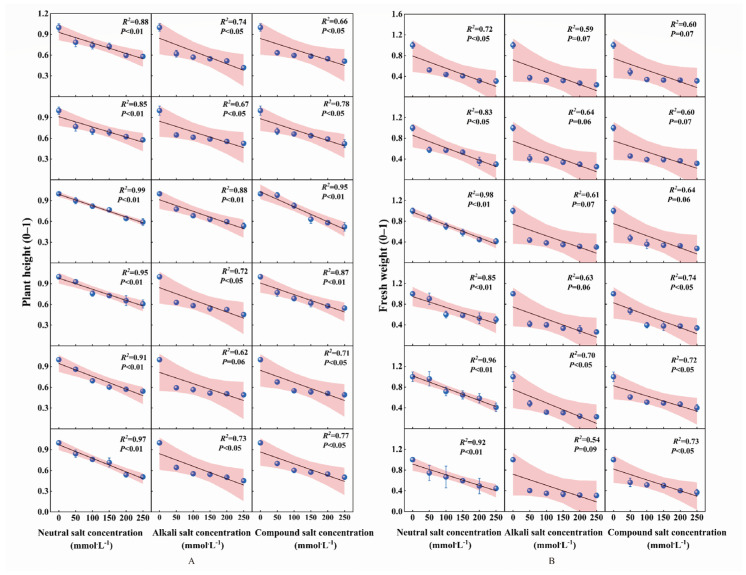
Linear regression analysis of salt tolerance index (STI) versus salt concentration gradients under different salt-stress conditions: (**A**) plant height (PH) STI versus concentration; (**B**) fresh weight (FW) STI versus concentration, the red shaded area represents the confidence interval of the regression fit, indicating the range of uncertainty around the regression line.

Based on the trait correlation heatmap shown in Figure A1, phenotypic traits with higher significance (PH, FW) were selected to quantify the salt tolerance threshold through linear regression of STI concentration relationships (Figure 5, Table A6). Under neutral salt stress, PH-STI regression models showed excellent fit (*R*^2^ = 0.85–0.99, all *p* < 0.01), producing genotype-specific thresholds of 215–410 mmol·L^−1^. Corresponding FW-STI models showed strong fits (*R*^2^ = 0.72–0.98, all *p* < 0.05) with thresholds ranging from 145 to 260 mmol·L^−1^, resulting in an average composite threshold of 235 mmol·L^−1^.

Model fitting under alkaline salt stress was inferior to that under neutral salt conditions. The PH-STI regression model exhibited moderate fit (*R*^2^ = 0.62–0.88; *p* < 0.05 for all except W05, which yielded *p* = 0.06), yielding a threshold range of 160–205 mmol·L^−1^. The FW-STI model exhibited a weaker fit (*R*^2^ = 0.54–0.70) with borderline significance (only W05 reached *p* < 0.05; *p*-values of 0.06–0.09 for the remaining samples). This model yielded a threshold range of 87–120 mmol·L^−1^ and an average composite threshold of 143 mmol·L^−1^.

Under combined stress, PH-STI maintained strong fits (*R*^2^ = 0.66–0.95, all *p* < 0.05; thresholds: 160–205 mmol·L^−1^), while FW-STI showed reduced performance (*R*^2^ = 0.60–0.74; W01/W02/W03: *p* = 0.06–0.07; others: *p* < 0.05) with thresholds of 120–165 mmol·L^−1^ (mean composite: 171 mmol·L^−1^).

**Figure 6:**
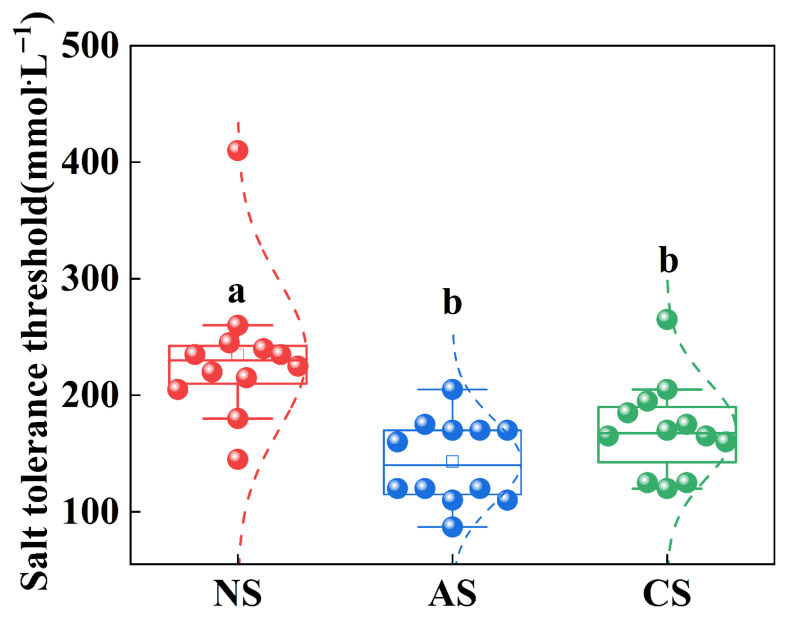
Differential distributions of salt-tolerance thresholds for PH and FW under contrasting salt-stress conditions: NS (neutral salt), AS (alkaline salt); CS (combined salt), different letters (a, b, c) indicate significant differences at *p* < 0.05 (Duncan’s multiple range test), while the same letter indicates no significant difference. The letters are assigned in descending order of the mean values.

Threshold distributions across all stresses followed a consistent pattern (Figure 6). ANOVA showed that thresholds were significantly higher under neutral stress than under alkaline or combined stresses. In contrast, there was no significant difference between alkaline and combined stresses. This indicates much greater growth inhibition under high-pH conditions.

### 2.7. Analysis of the Contribution of 21 Variables to Plant Height and Biomass in Wheat Seedlings Under Different Salt Stresses

**Figure 7:**
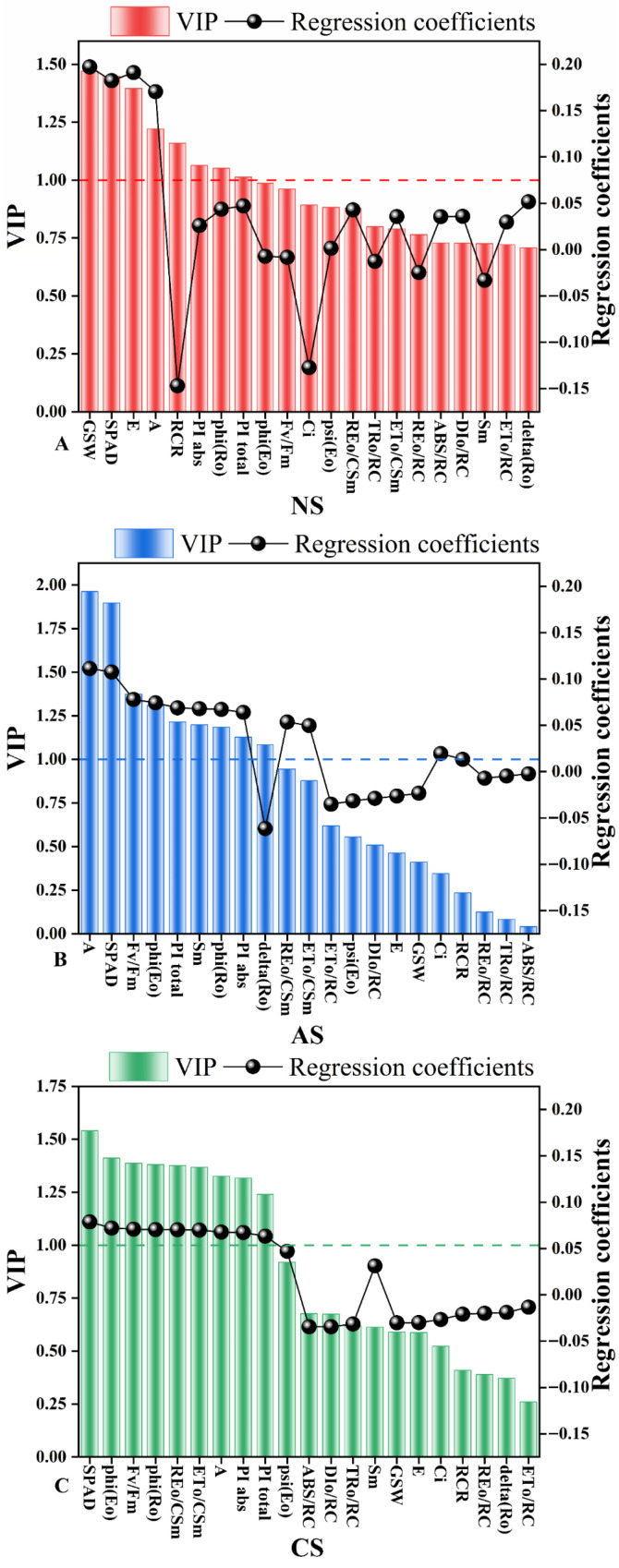
Variable importance in the projection (VIP) scores and regression coefficients (RCs) for 21 variables related to PH and biomass (FW, DW) in the partial least squares regression (PLSR) model for salt tolerance index of wheat seedlings under different salt stresses: (**A**) neutral salt stress, (**B**) alkaline salt stress, and (**C**) combined salt stress.

According to the PLSR results for PH and biomass under neutral salt stress (Figure 7A, Table S3), the VIP values for GSW, SPAD, E, A, RCR, PIabs, phi (Ro), and PItotal were all greater than 1. Among these, GSW had the highest VIP value (1.47), while RCR had the lowest regression coefficient (0.20). Under alkaline salt stress (Figure 7B, Table S3), only nine of the 21 variables had VIP values exceeding 1: A, SPAD, Fv/Fm, phi (Eo), PItotal, Sm, phi (Ro), PIabs, and delta (Ro). The variable A exerted the most significant influence, with a VIP of 1.96 and a regression coefficient of 0.11. Under combined salt stress (Figure 7C, Table S3), the set of variables with VIP > 1 differed from those in other treatments, except for REo/CSm and ETo/CSm. SPAD was identified as the most influential variable (VIP = 1.54, RCs = 0.08). In contrast, ETo/RC was the least significant, with the lowest VIP score (0.26) and a regression coefficient of −0.01.

### 2.8. Redundancy Analysis of Salt Concentration, Stress Ions, and Wheat Traits Under Salt Stress

**Figure 8:**
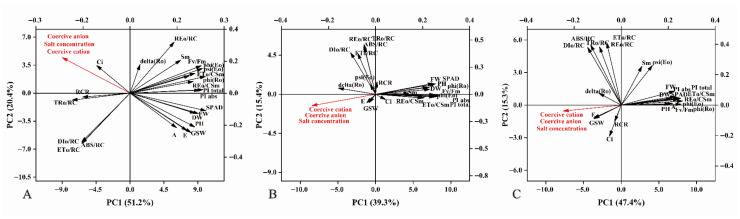
Redundancy analysis (RDA) of seedling traits under different salt stress conditions with respect to salt concentration and stress ions. (**A**) shows neutral salt stress, (**B**) shows alkaline salt stress, and (**C**) shows combined salt stress. Red arrows indicate environmental variables (salt concentration, stress ions), the black arrows indicate the parameters used to assess the seedlings. The dominant stress cation was sodium ion (Na^+^). Under neutral salt stress, the primary anions were sulfate (SO_4_^2−^) and chloride (Cl^−^); under alkaline salt stress, they were carbonate (CO_3_^2−^) and bicarbonate (HCO_3_^−^); under composite salt stress, anions included SO_4_^2−^, Cl^−^, CO_3_^2−^, and HCO_3_^−^.

Under neutral salt stress conditions, redundancy analysis (RDA) revealed that the first and second principal components (PC1 and PC2) explained 51.2% and 20.4% of the variation, respectively. Salt concentration and stress ions, including chloride (Cl^−^) and sulfate (SO_4_^2−^), were significantly linked to various seedling traits. Salt concentration, sodium ions (Na^+^), chloride ions (Cl^−^), and sulfate ions (SO_4_^2−^) showed significant negative correlations with pH, A, and GSW. In contrast, they were positively correlated with Ci and RCR (Figure 8A).

Under alkaline salt stress, RDA showed that principal components 1 and 2 accounted for 39.3% and 15.4% of the variation, respectively. Carbonate (CO_3_^2−^) and bicarbonate (HCO_3_^−^) ions were negatively associated with traits such as FW and SPAD. Additionally, these environmental variables displayed positive correlations with E and GSW (Figure 8B).

As shown in Figure 8C, RDA revealed that principal components PC1 and PC2 accounted for 47.4% and 15.3% of the variation, respectively. Salt concentration and stress ions, including chloride (Cl^−^), sulfate (SO_4_^2−^), carbonate (CO_3_^2−^), and bicarbonate (HCO_3_^−^), were linked to seedling traits. Among these, salt concentration and stress ions were negatively related to traits such as Sm, FW, SPAD, and Fv/Fm, whereas they were positively related to RCR and GSW.

### 2.9. Slope of Salt Stress Response in Wheat Seedlings of Different Genotypes Under Various Salt Stress Conditions

Based on the results shown in Figure 6, as stress intensity increased, FW and DW decreased across all genotypes, with W02 and W06 showing relatively less reduction in growth. To better measure salt tolerance, this study used the slope of biomass response to salt stress (relative to stress level) as a key indicator. A smaller absolute value of this slope signals stronger salt tolerance. The analysis in Table 1 showed significant differences in salt tolerance among genotypes, with effects of salt stress type. Under neutral salt stress, W06 showed the strongest tolerance, with the smallest absolute slope values for both FW and DW, followed by W02 and W04, while W01, W03, and W05 were less tolerant. Under alkaline salt stress, W02 performed best, with W03 and W06 also maintaining strong dry matter accumulation capacity. Under combined salt stress conditions, W02 and W06 exhibited the highest overall stress resistance, with a notably smaller decrease in dry weight than the other genotypes (Table 3).

### 2.10. Construction of Multivariate Linear Regression Models for Biomass (FW, DW) and PH and VIP > 1 Traits Under Different Salt Stresses

Under different salt stress conditions, multivariate linear models were developed for FW, DW, and PH using traits with VIP values > 1. The absolute values of the standardized regression coefficients for each trait indicate the strength of the association with the physiological parameter, with negative values indicating inhibitory effects and positive values indicating promoting effects.

Under neutral salt stress, the *R*^2^ of the multivariate linear models ranged from 0.727 to 0.849, and all models were statistically significant (*p* < 0.001). For FW, phi (Ro) had the most important positive influence, with a standardized regression coefficient of 1.248. In the DW model, E had the highest coefficient (0.528). For PH, phi (Ro) had the most substantial adverse effect (−25.695), while E demonstrated the most potent positive effect (31.735) (Table 4). Under alkaline salt stress, the *R*^2^ values of the models exceeded 0.900 for all parameters except DW (*R*^2^ = 0.461, *p* < 0.010), and all models were significant (*p* < 0.001). For FW, phi (Ro) had the most substantial positive influence (1.445), while phi (Eo) showed the most substantial negative influence (−1.149). In the DW model, phi (Ro) had the most significant absolute standardized regression coefficient (−0.187). For PH, phi (Ro) again exerted the greatest influence, with a coefficient of −87.301 (Table 5). Under combined salt stress, the *R*^2^ values of the models ranged from 0.528 to 0.918, and all *p*-values were <0.001. In the FW model, phi (Eo) exhibited the most substantial adverse effect (−0.599). For DW, phi (Ro) had the highest coefficient (0.170). In the PH model, phi (Ro) showed the most substantial adverse effect (−21.305), while Fv/Fm had the most potent positive effect (13.386) (Table 6).

## 3. Discussion

### 3.1. Seedling-Related Traits Respond Significantly to Salt Concentration, Genotype, and Salt Type

Three-way ANOVA revealed that SC, G, and ST significantly influenced all seedling-related traits, with the three-way interaction (SC × G × ST) significantly affecting photosynthetic parameters (Table 1). This finding aligns with Shin et al. (2020), who demonstrated that salt stress significantly suppressed chlorophyll fluorescence parameters, growth indices, phytochemical composition, and antioxidant activity in lettuce [14]. Notably, among the gas-exchange parameters, only transpiration rate (E) and gas-exchange conductance (GSW) showed significant effects under the SC × G × ST interaction. In contrast, no significant interaction effects were observed for relative chlorophyll content (SPAD). This contrasts with the findings of Tang et al. (2018), who reported that salt stress induces significant changes in plant parameters, including SPAD, A, GSW, E, and Fv/Fm [15]. This discrepancy may stem from differences in the mechanisms underlying seedling photosynthetic responses to salt stress, encompassing both stomatal and non-stomatal limitations. For instance, Pflüger et al. (2024) indicated that GSW is the most sensitive drought parameter in wheat, with progressive stomatal closure limiting photosynthesis when GSW falls below genotype-specific thresholds [16]. A similar mechanism may operate under salt stress. Analysis of the STI distribution heatmap revealed that A, SPAD, and Fv/Fm values for all genotypes negatively correlated with salt concentration across different salt types. This indicates that the present findings do not contradict Tang et al.’s observations but rather reveal a more complex interaction between stress type, genotype, and concentration. In summary, the SC × G × ST interaction significantly influenced GSW and E, highlighting the importance of stomatal limitation in complex salt-gene-environment interactions. The absence of interaction effects on SPAD values suggests other factors, such as main effects or simple interactions, may regulate chlorophyll degradation.

### 3.2. Partial Least Squares Structural Equation Modeling Reveals Significant Differences in Seedling Photosynthetic Responses Under Different Salt Types

Based on the Pearson correlation coefficient and Mantel analysis results, the key traits required to construct the PLS-SEM were selected. In this model, BM, the ultimate product of photosynthetic accumulation, is designated as the dependent variable, while the remaining traits serve as latent variables that influence BM. The analysis indicates that the influence pathways of these latent variables on BM differ significantly under various salt stress conditions (Figure 4, Table 2 and Table A4). The reduction in seedling biomass accumulation is significantly affected by declines in photosynthesis, which are closely related to both stomatal and non-stomatal limitations. Under short-term, low-concentration salt stress, seedlings primarily experience osmotic stress [16,17,18]. At this stage, the bZIP gene family member *TabZIP60* and the NAC gene family member *TaNAC5D-2* are activated, inducing stomatal closure by regulating ABA signaling to reduce water loss [19,20]. The photosynthetic decline during this phase is mainly characterized by stomatal limitation, specifically manifested by a simultaneous decrease in A, GSW, and Ci [16,17,18]. However, with prolonged stress duration and increased salt concentration, ion toxicity and oxidative damage gradually become dominant. This leads to a shift in the photosynthetic limitation of seedlings from stomatal to non-stomatal limitation. During this process, WRKY and DREB/CBF family genes play crucial roles in regulating chloroplast ROS homeostasis [21,22]. The typical indicators of non-stomatal limitation are an increase or no change in Ci, accompanied by a significant decrease in the maximum photochemical efficiency of photosystem II (Fv/Fm) [16,17,18]. The specific performances under different salt stress conditions are as follows:

Under neutral salt stress, the PLS-SEM model encompassed key gas exchange parameters (A, GSW, E, Ci). The model reveals that while CFPS has no significant direct effect on BM, all other pathways do (Figure 4B). This finding aligns with the photosynthetic response characteristics of seedlings and concurs with Ma et al. (2025), who observed in cotton under 150 mmol·L^−1^ NaCl stress that enhanced stomatal limitation synergized with reduced non-stomatal limitation to maintain stable photosynthetic rates and improve water use efficiency [23]. The STI distribution heatmap further indicates that both stomatal and non-stomatal limitations coexist under neutral salt stress. Specifically, at lower salt concentrations (100 mmol·L^−1^), minimal changes in gas exchange and chlorophyll fluorescence parameters suggest stomatal limitation predominates; As salt concentration increased, both gas exchange parameters (A, E, GSW) and chlorophyll fluorescence parameters (PIabs, PItotal) decreased significantly, indicating that non-stomatal limiting factors gradually became dominant, with both factors exhibiting synergistic effects.

Compared with neutral salt stress, the PLS-SEM model under alkaline salt stress showed distinct differences in the BM pathway: only Chl C had a direct, significant effect on BM (Figure 4C). Heatmap analysis revealed substantial declines in gas exchange parameters (E, A, GSW) and multiple chlorophyll fluorescence parameters (PIabs, PItotal, phi (Ro), ETo/CSm, Fv/Fm). This indicates that prolonged alkaline stress induces accumulation of reactive oxygen species, damaging the PSII reaction center and thereby reducing light-harvesting efficiency. Excess light energy dissipates via non-photochemical quenching (NPQ), ultimately reducing photochemical efficiency and electron transport rates, triggering chlorophyll degradation and decreased BM [24].

Under combined salt stress, the PLS-SEM model pathway for BM differed from those under neutral and alkaline salt stress, with Chl C again exhibiting a direct and significant effect on BM (Figure 4D). The STI heatmap indicates an interaction between stomatal and non-stomatal factors at the low concentration (50 mmol·L^−1^) on day 7 (Figure 1). However, as the stress concentration increases, the steep decline in related traits suggests that non-stomatal limitation gradually becomes dominant. This process involves reactive oxygen species-mediated damage to the chloroplast membrane system, activation of chlorophyll degradation enzymes, and inhibition of key chlorophyll biosynthesis enzymes, ultimately leading to significantly reduced SPAD values and decreased BM accumulation. This phenomenon aligns with the findings of Shi et al. (2021) [25].

In summary, the photosynthetic responses of wheat seedlings exhibit significant differences under various salt stress conditions. This study reveals that as stress intensity increases, all salt types exhibit a universal shift in photosynthetic limiting mechanisms—from stomatal limitation during low-stress periods to non-stomatal limitation during high-stress periods. To more accurately determine whether stomatal closure becomes the primary limiting factor in seedlings under stress, future research should integrate comprehensive analyses including ultrastructural observations of leaf cells, measurements of key photosynthetic enzyme activities, and assessments of reactive oxygen species levels.

### 3.3. Multivariate Analysis of Trait Contributions to Seedling Biomass and Height Under Salt Stress

Based on STI and SDI analysis results, the FW, DW, and PH of seedlings showed significant negative correlations with salt concentration (Figure 1). Redundancy analysis further revealed that FW, DW, and PH also negatively correlated with stress ion content. This finding aligns with He et al. (2023), who reported significant disruption of ecological profiles in roots, stems, and leaves of *Lycium barbarum* seedlings under varying Pb stress concentrations [26].

To identify the most influential indicators affecting seedling phenotypes (FW, DW, PH) under salt stress, we first calculated VIP scores for each indicator using PLS. Generally, indicators with VIP > 1 are considered to contribute more [27]. Our analysis showed that the traits with VIP values greater than 1 varied across the three salt stress types, suggesting possible links to distinct injury mechanisms (Figure 7). To further compare the contribution of these traits to different salt stresses, we quantified them using standardized regression coefficients from a multiple linear regression model [28].

Simulation results indicate that under neutral salt stress, the trait most significantly affecting FW is phi (Ro) (coefficient 1.248) (Table 4). As a key parameter reflecting PSI function, phi (Ro) has been shown in previous studies to significantly decrease under stress conditions, indicating that neutral salt stress primarily inhibits electron transport on the PSI acceptor side [29]. The indicator exerting the most substantial positive influence on both DW and PH was E (transpiration rate). Consistent with STI results, both E and GSW decreased as salt concentration increased, indirectly confirming that neutral salt stress mainly affects seedling growth through osmotic stress [30].

Under alkaline salt stress, Fv/Fm had the most significant impact on FW (Table 5). STI results showed that Fv/Fm decreased with increasing salt concentration, indicating that alkaline salt stress disrupted the chlorophyll fluorescence system [31]. The effect of phi (Ro) on DW further supports this conclusion. In the PH regression model, phi (Eo) was a significant predictor, along with Fv/Fm and phi (Ro). As a parameter indicating the maximum potential photochemical efficiency of the PSII reaction center, the notable contribution of phi (Eo) demonstrates that alkaline salt stress also damages PSII [32].

Under combined salt stress, both phi (Ro) and phi (Eo) significantly affected FW, with phi (Ro) having the most impact on DW (Table 6). Moreover, phi (Ro), phi (Eo), and Fv/Fm all notably contributed to PH, indicating that combined salt stress displays traits of both neutral and alkaline salt stress [33].

### 3.4. Salt Tolerance Thresholds of Seedlings with Different Genotypes and Their Comprehensive Evaluation

Our analysis established linear regression equations relating seedling STI (PH and FW) to salt concentration to determine salt tolerance thresholds. Significant differences emerged among salt stress types. Distribution heatmaps indicated that alkaline and combined salt stress led to notably steeper concentration-dependent declines in STI compared to neutral salt stress (Figure 5 and Figure 6). This pattern means that seedlings sustained substantially greater damage under alkaline and combined salt stress than under neutral salt conditions. These findings align with those of Guo et al. (2017), who emphasized that neutral salt stress lacks the high pH component inherent in alkaline and combined salts [34].

To thoroughly assess wheat seedling biomass across genotypes, we developed linear regression equations using biomass trend curves and calculated salt-stress response slopes. Under neutral salt stress, W06 showed the greatest tolerance, as indicated by the salt-stress response slope (Table 3). Analysis of phenotypic characteristics (PH, RCR) using STI and SDI confirmed that W06’s STI was higher than that of other genotypes across all stress levels, demonstrating its superior performance. Regarding the key traits most significantly affecting DW and FW, phi (Ro) and E (transpiration rate) were identified. Further analysis of E’s STI and SDI revealed that plants reduce water loss by lowering E under salt stress. W06 exemplified this response, maintaining lower E values compared to other varieties [35]. Additionally, a higher phi (Ro) value indicates more efficient electron transport on the PSI acceptor side and a more complete photosynthetic electron transport chain. STI results showed that W06’s phi (Ro) remained relatively stable across varying concentrations, suggesting that its photosynthetic electron transport was minimally affected by neutral salt stress [36].

Under alkaline salt stress, the traits most significantly affecting biomass were phi (Eo) and phi (Ro). Combined STI and SDI analyses showed that W02 exhibited less variation in both phi (Eo) and phi (Ro) than other genotypes (Figure 1). Since phi (Eo) reflects PSII’s efficiency in converting absorbed light energy into electron transport, its stability in W02 indicates better maintenance of photosynthetic function under stress. These results are consistent with W02’s performance in salt stress response slope analysis [37].

Under combined salt stress, the salt stress response slope indicated that both W02 and W06 performed better overall (Table 3). Given that phi (Eo) and phi (Ro) are crucial for biomass, and these parameters exhibited minimal fluctuations in W02 and W06 during STI analysis, the stability of these key photosynthetic parameters explains the increased resistance of these two genotypes under combined salt stress.

## 4. Materials and Methods

### 4.1. Plant Materials and Growth Conditions

Six spring wheat genotypes were obtained from the Inner Mongolia Academy of Agricultural and Animal Sciences for this study. The detailed information is shown in Table 7.

This experiment was conducted at the Institute of Crop Science, Inner Mongolia Academy of Agricultural and Animal Husbandry Sciences (111.669° E, 40.773° N) in Hohhot, China, between 2024 and 2025. Uniform wheat seeds were surface-sterilized with 75% ethanol for 15 min, then thoroughly rinsed with distilled water. Subsequently, the seeds were soaked in distilled water for 10 h. A total of 300 seeds were then placed on sterile filter paper in Petri dishes and germinated under controlled conditions: 25 ± 2 °C, 60–70% relative humidity, and a 12/12 h light/dark photoperiod. Upon reaching a height of 3–5 cm, uniform seedlings were selected and transplanted into a hydroponic system [13].

Hoagland nutrient solution was used to fulfill the nutritional requirements of the hydroponic system plants. Uniformly grown seedlings were transplanted into rectangular polyethylene containers (dimensions: 40 cm long × 30 cm wide × 23 cm high), each filled with 4.0 L of Hoagland solution for hydroponic cultivation. To avoid light exposure, the root systems were kept in darkness throughout the entire growth period. Environmental conditions were maintained at 25 ± 2 °C, with relative humidity kept between 60% and 70%, and a 12 h light/12 h dark cycle was used [38,39,40].

When seedlings reached the two-leaf stage, different types and levels of salt stress treatments were applied. Specific salt stress types and concentrations were set as follows (Table 2): Neutral salt stress (NaCl:Na_2_SO_4_ = 1:1 molar ratio): 0, 50, 100, 150, 200, 250 mmol·L^−1^; Alkali salt stress (Na_2_CO_3_:NaHCO_3_ = 1:1): 0, 50, 100, 150, 200, 250 mmol·L^−1^; Combined salt stress (NaCl:Na_2_SO_4_:Na_2_CO_3_:NaHCO_3_ = 1:1:1:1): 0, 50, 100, 150, 200, 250 mmol·L^−1^. Detailed treatment specifications are provided in Table 8 [41,42,43]. Each treatment included four biological replicates. Measurements of relevant physiological indicators began on day 6 of the stress treatment [44,45].

All seedlings were randomly arranged in the growth chamber to eliminate positional effects. Each treatment consisted of 4 biological replicates, with 3 seedlings per replicate. The stress concentration range was determined based on preliminary experiments: within 0–250 mmol·L^−1^, spring wheat seedlings exhibited significant but non-lethal stress effects, accompanied by gradient-dependent photosynthetic responses. Physiological traits stabilized after 5 days of stress treatment; thus, the 6th day was selected as the sampling time point for investigating photosynthetic adaptation.

### 4.2. Experimental Method

#### 4.2.1. Plant Height

Seedling height was measured and recorded for all plants after 6 days of stress treatment [46].

#### 4.2.2. Relative Chlorophyll Content

On the sixth day of stress treatment, chlorophyll content was assessed in the youngest fully expanded leaves of four randomly sampled seedlings per replicate using an SPAD-502 Plus meter (Konica Minolta Inc., Tokyo, Japan). Three measurements were taken parallel to the midrib on the adaxial leaf surface, avoiding primary veins (located 1/3 from the leaf base), with consistent positioning across all treatments.

#### 4.2.3. Chlorophyll Fluorescence Parameters

They were measured using a Handy PEA plant efficiency analyzer (Hansatech Instruments Ltd., King’s Lynn, UK). Following a 30 min dark-adaptation period, chlorophyll measurements were performed on the evening of the sixth day after the stress treatment. For each treatment, the same leaf from each seedling was measured three times between 6:30 p.m. and 12:00 a.m. Specific measurements are provided in Table 9 [47].

#### 4.2.4. Gas Exchange Parameters

Gas exchange parameters were measured using the LI-6800 Portable Photosynthesis System (LI-COR Inc., Lincoln, NE, USA), with key indicators including net photosynthetic rate (A), transpiration rate (E), stomatal conductance (GSW), and intercellular carbon dioxide concentration (Ci). Measurements started on the 7th day after stress application and were taken between 8:00 a.m. and 5:00 p.m. on a clear day. For each seedling across all treatments, three replicate measurements were recorded. The instrument was set to an airflow rate of 1500 mL·min^−1^, a CO_2_ concentration of 500 μmol·mol^−1^, a relative humidity of 60%, and a photosynthetic photon flux density (PPFD) of 800 μmol·m^−2^·s^−1^ provided by the built-in light source [48].

#### 4.2.5. Biomass

After measuring the gas exchange parameters, the seedlings were placed in self-sealing bags and stored frozen. For each treatment, all seedling samples were pooled to determine the fresh weight of individual plants using a 1/10,000 analytical balance (ME204, METTLER TOLEDO Inc., Greifensee, Switzerland). After measuring fresh weight, the samples were dried in a WFO-520 forced-air, temperature-controlled drying oven (okyo Physical and Chemical Instrument Co., Ltd., Tokyo, Japan) using a two-step protocol: initial drying at 105 °C for 30 min, followed by drying at 80 °C until constant weight was achieved. The dry weight was then measured with the same 1/10,000 analytical balance [49].

#### 4.2.6. Salt Tolerance Threshold, Salt Tolerance Index, and Salt Damage Index

A linear regression model was fit to connect the salt-tolerance index (STI) to increasing salt concentrations, based on seedling height and fresh weight data. The salt-tolerance threshold was defined as the concentration (X) where the fitted regression line predicted STI = 0.5 [36,46,47,48,49,50,51].


(1) $$\mathrm{STI}=\frac{\mathrm{value}\;\mathrm{of}\;\mathrm{trait}\;\mathrm{under}\;\mathrm{stress}\;\mathrm{condition}}{\mathrm{value}\;\mathrm{of}\;\mathrm{trait}\;\mathrm{under}\;\mathrm{controlled}\;\mathrm{condition}}$$

The Salt Damage Index (SDI) is used to measure changes in plant traits caused by salt stress [46,51].


(2) SDI=1−STI

Indicators significantly correlated (Pearson, *p* < 0.05) with both dry weight and fresh weight were selected and grouped into four physiological categories: (1) biomass, (2) chlorophyll content, (3) gas exchange parameters, and (4) chlorophyll fluorescence parameters for the model construction.

#### 4.2.7. Root-Crown Ratio [52]


(3) $$\text{Root-crown ratio}=\frac{\text{below-ground dry weight}}{\text{above-ground dry weight}}$$

### 4.3. Statistical Analysis of Data

Data were analyzed and visualized using several software packages. Microsoft Excel 2016 was used for data organization and computation. Statistical analyses, including three-way ANOVA and linear regression, were performed using IBM SPSS Statistics 22. OriginPro 2022 and the R (4.4.2) programming language were used for data visualization, such as heatmaps, correlation networks, and redundancy analysis. Structural equation modeling and path coefficient analysis were conducted in SmartPLS 4.0. Variable importance in projection (VIP) and regression coefficients in partial least squares regression were calculated using SIMCA 14.1. Statistical significance was set at *p* < 0.05, with higher significance levels denoted as *p* < 0.01 and *p* < 0.001.

## 5. Conclusions

Three-way ANOVA revealed that interactions among salt type, genotype, and concentration significantly influenced seedling traits, particularly gas exchange (E, GSW) and chlorophyll fluorescence parameters (e.g., Fv/Fm, phi (Eo), PIabs). Redundancy analysis showed that environmental factors, especially stress ions, were negatively correlated with biomass, net photosynthetic rate, and chlorophyll content.

Using Pearson correlation coefficients and network heatmaps, PLS-SEM was applied to identify distinct photosynthetic response patterns under different levels of salt stress. Under neutral salt stress, gas exchange parameters and chlorophyll content had significant direct effects on biomass, while chlorophyll fluorescence parameters directly affected only chlorophyll content. In contrast, under alkaline salt stress, only chlorophyll content exhibited a substantial direct effect on biomass. Furthermore, under alkaline stress, gas exchange parameters directly influenced chlorophyll fluorescence parameters, whereas the interrelations among chlorophyll fluorescence parameters themselves remained similar to those under neutral stress. Under combined salt stress, chlorophyll content remained the only factor with a significant direct effect on biomass.

**Figure 9:**
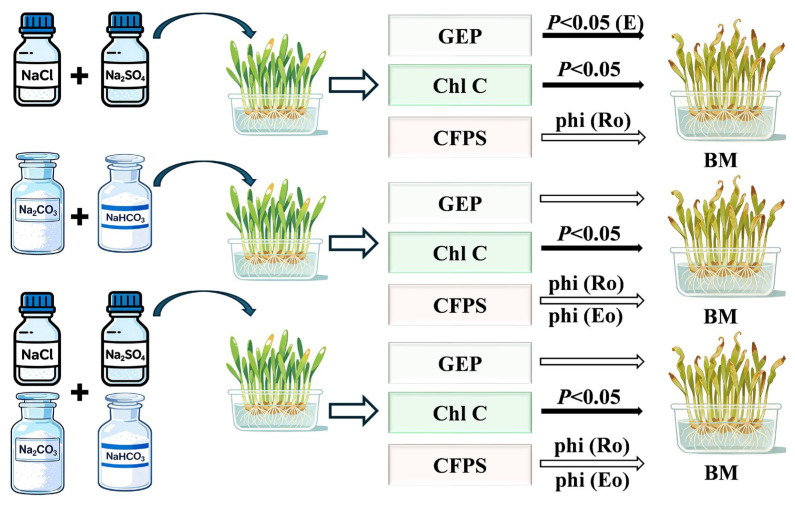
Differences in the effects of seedling photosynthesis on biomass under different types of salt stress. Gas exchange parameters (GEP), chlorophyll content (Chl C), chlorophyll fluorescence parameters (CFPS) and biomass (BM) were analyzed, where black filled arrow (*p* < 0.05) indicate paths with significant direct effects on biomass in the structural equation model, white filled arrows indicate non-significant paths, and transpiration rate (E), quantum yield of reduction at the terminal electron acceptor of PSI (phi (Ro)), quantum yield of electron transport (phi (Eo)) are indicators that significantly affect biomass in the multiple regression analysis.

Based on the partial least squares models established for FW, DW, and PH and their corresponding VIP values, we further determined the standardized regression coefficients for each trait through multiple linear regression. The results indicated significant differences in the key determinants of biomass under different stress conditions (Figure 9). Under neutral salt stress, phi (Ro) had the greatest positive effect on FW, while E had the greatest positive effect on DW. For PH, phi (Ro) had the most pronounced adverse effect, whereas E demonstrated the most potent positive effect. Under alkaline salt stress, phi (Ro) exerted the most significant positive influence on FW, whereas phi (Eo) produced the most pronounced adverse effect; additionally, phi (Ro) demonstrated the most potent adverse effects on both DW and PH. Under combined salt stress, the negative influence of phi (Eo) was most pronounced in the FW model; phi (Ro) exhibited the highest regression coefficient (positive effect) in the DW model; while in the PH model, the adverse impact of phi (Ro) was most significant, and Fv/Fm demonstrated the most potent positive effect.

Finally, the analysis of salt tolerance thresholds across genotypes under varying stress levels confirmed that alkaline and combined salt stresses caused significantly greater damage to seedlings than neutral salt stress. By integrating STI, SDI, and stress response slopes, the evaluation of genotype performance indicated that W06 showed strong tolerance to neutral salt stress. Whereas W02 exhibited greater tolerance to alkaline salt stress, under which W06 performed relatively poorly. Under combined salt stress, both W02 and W06 displayed the highest overall stress resistance among the genotypes tested.

## Figures and Tables

**Table 1:** Three-way ANOVA examining the effects of salt type (ST), genotype (G), and salt concentration (SC) on physiological traits in wheat seedlings. Significant effects (*p* < 0.05) are highlighted in bold.

Indicator Name	ST		G		SC		ST × G		ST × SC		G × SC		ST × G × SC	
	*F*-Value	*p*-Value	*F*-Value	*p*-Value	*F*-Value	*p*-Value	*F*-Value	*p*-Value	*F*-Value	*p*-Value	*F*-Value	*p*-Value	*F*-Value	*p*-Value
PH	83.60	***p* < 0.01**	53.21	***p* < 0.01**	431.10	***p* < 0.01**	2.02	***p* < 0.05**	5.87	***p* < 0.01**	3.58	***p* < 0.01**	0.80	0.833
FW	69.93	***p* < 0.01**	24.18	***p* < 0.01**	234.08	***p* < 0.01**	3.36	***p* < 0.01**	4.94	***p* < 0.01**	1.55	***p* < 0.05**	0.39	1.000
DW	14.86	***p* < 0.01**	17.52	***p* < 0.01**	37.10	***p* < 0.01**	0.84	0.593	0.64	0.779	0.55	0.963	0.26	1.000
RCR	73.11	***p* < 0.01**	1.98	0.081	14.84	***p* < 0.01**	0.44	0.927	3.26	***p* < 0.01**	0.76	0.789	0.51	0.998
E	15.35	***p* < 0.01**	11.08	***p* < 0.01**	36.20	***p* < 0.01**	2.07	***p* < 0.05**	8.38	***p* < 0.01**	3.21	***p* < 0.01**	2.21	***p* < 0.01**
A	12.23	***p* < 0.01**	2.28	***p* < 0.05**	47.88	***p* < 0.01**	0.60	0.815	0.62	0.793	0.77	0.778	0.16	1.000
Ci	0.34	0.715	0.54	0.748	0.61	0.695	0.50	0.887	0.52	0.877	0.35	0.999	0.31	1.000
GSW	9.31	***p* < 0.01**	9.63	***p* < 0.01**	34.12	***p* < 0.01**	2.65	***p* < 0.01**	8.00	***p* < 0.01**	3.49	***p* < 0.01**	2.13	***p* < 0.01**
Fv/Fm	569.18	***p* < 0.01**	14.21	***p* < 0.01**	279.08	***p* < 0.01**	3.89	***p* < 0.01**	43.90	***p* < 0.01**	6.69	***p* < 0.01**	5.66	***p* < 0.01**
Sm	52.61	***p* < 0.01**	8.55	***p* < 0.01**	32.48	***p* < 0.01**	4.79	***p* < 0.01**	9.01	***p* < 0.01**	3.65	***p* < 0.01**	3.11	***p* < 0.01**
ABS/RC	8.50	***p* < 0.01**	1.93	0.090	6.09	***p* < 0.01**	4.29	***p* < 0.01**	3.68	***p* < 0.01**	2.40	***p* < 0.01**	2.64	***p* < 0.01**
DIo/RC	17.71	***p* < 0.01**	2.74	***p* < 0.05**	12.95	***p* < 0.01**	4.71	***p* < 0.01**	4.39	***p* < 0.01**	3.22	***p* < 0.01**	2.19	***p* < 0.01**
TRo/RC	9.16	***p* < 0.01**	2.70	***p* < 0.05**	5.12	***p* < 0.01**	2.71	***p* < 0.01**	2.49	***p* < 0.01**	1.67	***p* < 0.05**	1.30	0.093
ETo/RC	0.27	0.762	3.70	***p* < 0.01**	2.58	***p* < 0.05**	4.05	***p* < 0.01**	2.07	***p* < 0.05**	2.31	***p* < 0.01**	2.12	***p* < 0.01**
REo/RC	2.97	0.053	2.68	***p* < 0.05**	4.31	***p* < 0.01**	2.82	***p* < 0.01**	1.70	0.079	2.10	***p* < 0.01**	2.06	***p* < 0.01**
psi (Eo)	32.22	***p* < 0.01**	2.70	***p* < 0.05**	13.55	***p* < 0.01**	2.52	***p* < 0.01**	2.59	***p* < 0.01**	2.94	***p* < 0.01**	1.93	***p* < 0.01**
phi (Eo)	945.44	***p* < 0.01**	11.75	***p* < 0.01**	403.68	***p* < 0.01**	5.07	***p* < 0.01**	59.59	***p* < 0.01**	5.18	***p* < 0.01**	5.31	***p* < 0.01**
delta (Ro)	11.53	***p* < 0.01**	3.64	***p* < 0.01**	6.04	***p* < 0.01**	3.41	***p* < 0.01**	2.81	***p* < 0.01**	3.16	***p* < 0.01**	3.04	***p* < 0.01**
phi (Ro)	645.56	***p* < 0.01**	17.85	***p* < 0.01**	369.27	***p* < 0.01**	4.08	***p* < 0.01**	38.03	***p* < 0.01**	4.93	***p* < 0.01**	5.32	***p* < 0.01**
ETo/CSm	1249.68	***p* < 0.01**	32.75	***p* < 0.01**	448.81	***p* < 0.01**	10.82	***p* < 0.01**	66.89	***p* < 0.01**	10.23	***p* < 0.01**	7.52	***p* < 0.01**
REo/CSm	1157.45	***p* < 0.01**	25.63	***p* < 0.01**	559.73	***p* < 0.01**	7.93	***p* < 0.01**	63.06	***p* < 0.01**	12.77	***p* < 0.01**	10.36	***p* < 0.01**
PIabs	365.69	***p* < 0.01**	9.74	***p* < 0.01**	168.69	***p* < 0.01**	5.51	***p* < 0.01**	21.97	***p* < 0.01**	2.77	***p* < 0.01**	2.27	***p* < 0.01**
PItotal	165.84	***p* < 0.01**	12.94	***p* < 0.01**	125.89	***p* < 0.01**	2.83	***p* < 0.01**	10.61	***p* < 0.01**	4.45	***p* < 0.01**	1.36	0.064
SPAD	631.50	***p* < 0.01**	7.46	***p* < 0.01**	353.99	***p* < 0.01**	2.41	***p* < 0.01**	29.02	***p* < 0.01**	1.17	0.268	0.84	0.765

Note: Values with *p* < 0.05 are presented in bold; exact *p* -values are not shown.

**Table 2:** Effects of GEP, CFPS, and Chl C on BM under various salt stress conditions, as shown by structural equation modeling.

Process	Indicator Name	Direct Impact	Indirect Impact	Total Impact
Control	**GEP**			
	CFPS	0.341	NA	0.341
	Chl C	0.357	−0.071	0.286
	BM	−0.063	0.258	0.195
	**CFPS**			
	GEP	NA	NA	NA
	Chl C	−0.207	NA	−0.207
	BM	0.445	−0.077	0.368
	**Chl C**			
	GEP	NA	NA	NA
	CFPS	NA	NA	NA
	BM	0.372	NA	0.372
Neutral salt stress	**GEP**			
	CFPS	0.545	NA	0.545
	Chl C	0.577	0.234	0.811
	BM	0.421	0.390	0.811
	**CFPS**			
	GEP	NA	NA	NA
	Chl C	0.429	NA	0.429
	BM	0.121	0.171	0.292
	**Chl C**			
	GEP	NA	NA	NA
	CFPS	NA	NA	NA
	BM	0.400	NA	0.400
Alkaline salt stress	**GEP**			
	CFPS	0.913	NA	0.913
	Chl C	0.218	0.668	0.886
	BM	0.336	0.525	0.861
	**CFPS**			
	GEP	NA	NA	NA
	Chl C	0.731	NA	0.731
	BM	−0.273	0.639	0.366
	**Chl C**			
	GEP	NA	NA	NA
	CFPS	NA	NA	NA
	BM	0.873	NA	0.873
Combined salt stress	**GEP**			
	CFPS	0.877	NA	0.877
	Chl C	0.434	0.488	0.922
	BM	0.226	0.631	0.857
	**CFPS**			
	GEP	NA	NA	NA
	Chl C	0.556	NA	0.556
	BM	−0.086	0.426	0.340
	**Chl C**			
	GEP	NA	NA	NA
	CFPS	NA	NA	NA
	BM	0.766	NA	0.766

Note: The bolded abbreviations represent the initial variables for each path in the structural equation model.

**Table 3:** Slope of seedling biomass response to salt stress under different salt stress conditions.

Salt Type	Genotype	Traits	Linear Regression Equation	*R* ^2^	Salt Stress Response Slope
Neutral salt stress	W01	FW	y = −0.0016x + 0.5408	0.7235	−0.0016
		DW	y = −9 × 10^−5^x + 0.0517	0.8735	−9 × 10^−5^
	W02	FW	y = −0.0011x + 0.3932	0.8301	−0.0011
		DW	y = −8 × 10^−5^x + 0.0436	0.9775	−8 × 10^−5^
	W03	FW	y = −0.0015x + 0.5827	0.9751	−0.0015
		DW	y = −7 × 10^−5^x + 0.0512	0.9693	−7 × 10^−5^
	W04	FW	y = −0.0013x + 0.5831	0.8459	−0.0013
		DW	y = −9 × 10^−5^x + 0.0555	0.9278	−9 × 10^−5^
	W05	FW	y = −0.0015x + 0.6449	0.9599	−0.0015
		DW	y = −8 × 10^−5^x + 0.0575	0.8331	−8 × 10^−5^
	W06	FW	y = −0.0012x + 0.5364	0.9206	−0.0012
		DW	y = −6 × 10^−5^x + 0.0495	0.8238	−6 × 10^−5^
Alkali salt stress	W01	FW	y = −0.0016x + 0.4888	0.5909	−0.0016
		DW	y = −9 × 10^−5^x + 0.0547	0.9367	−9 × 10^−5^
	W02	FW	y = −0.0011x + 0.3422	0.6403	−0.0011
		DW	y = −7 × 10^−5^x + 0.0450	0.9497	−7 × 10^−5^
	W03	FW	y = −0.0013x + 0.4434	0.6067	−0.0013
		DW	y = −5 × 10^−5^x + 0.0509	0.9706	−5 × 10^−5^
	W04	FW	y = −0.0014x + 0.4595	0.6327	−0.0014
		DW	y = −7 × 10^−5^x + 0.0539	0.9374	−7 × 10^−5^
	W05	FW	y = −0.0017x + 0.4806	0.6963	−0.0017
		DW	y = −7 × 10^−5^x + 0.0571	0.9288	−7 × 10^−5^
	W06	FW	y = −0.0013x + 0.4220	0.5450	−0.0013
		DW	y = −5 × 10^−5^x + 0.0516	0.8460	−5 × 10^−5^
Combined salt stress	W01	FW	y = −0.0015x + 0.5082	0.6031	−0.0015
		DW	y = −9 × 10^−5^x + 0.0601	0.9187	−9 × 10^−5^
	W02	FW	y = −0.0010x + 0.3444	0.5957	−0.0010
		DW	y = −5 × 10^−5^x + 0.0456	0.9046	5 × 10^−5^
	W03	FW	y = −0.0014x + 0.4501	0.6442	−0.0014
		DW	y = −6 × 10^−5^x + 0.0512	0.9661	−6 × 10^−5^
	W04	FW	y = −0.0015x + 0.5094	0.7367	−0.0015
		DW	y = −4 × 10^−5^x + 0.0555	0.6915	−4 × 10^−5^
	W05	FW	y = −0.0012x + 0.5242	0.7187	−0.0012
		DW	y = −6 × 10^−5^x + 0.0594	0.7878	−6 × 10^−5^
	W06	FW	y = −0.0012x + 0.4807	0.7324	−0.0012
		DW	y = −4 × 10^−5^x + 0.0552	0.4274	−4 × 10^−5^

**Table 4:** Standardized regression coefficients of multivariate regression models for PH, FW, and DW based on VIP > 1 traits under neutral salt stress.

Traits	FW	DW	PH
*p*-value	*p* < 0.001	*p* < 0.001	*p* < 0.001
*R* ^2^	0.727	0.729	0.849
RCR	0.009	−0.012	−8.320
E	0.264	0.528	31.735
A	0.002	0	0.328
GSW	0.317	0.018	6.800
phi (Ro)	1.248	0.084	−25.695
PIabs	−0.025	−0.001	0.670
PItotal	0.008	0	−0.413
SPAD	0.012	0.001	0.137

**Table 5:** Standardized regression coefficients of multivariate regression models for PH, FW, and DW based on VIP > 1 traits under alkaline salt stress.

Traits	FW	DW	PH
*p*-value	*p* < 0.001	*p* < 0.010	*p* < 0.001
*R* ^2^	0.947	0.461	0.939
A	0.006	0.001	0.437
Fv/Fm	0.199	−0.018	−12.902
Sm	0	−6.13 × 10^−5^	−0.010
phi (Eo)	−1.149	0.099	42.676
delta (Ro)	−0.005	0	0.355
phi (Ro)	1.445	−0.187	−87.301
PIabs	0.059	−0.002	0.372
PItotal	−0.051	0.002	0.363
SPAD	0.008	0	0.091

**Table 6:** Standardized regression coefficients of multivariate regression models for PH, FW, and DW based on VIP > 1 traits under combined salt stress.

Traits	FW	DW	PH
*p*-value	*p* < 0.001	*p* < 0.001	*p* < 0.001
*R* ^2^	0.912	0.528	0.918
A	0.004	0	0.411
Fv/Fm	0.051	0.014	13.386
phi (Eo)	−0.599	−0.076	−20.481
phi (Ro)	0.492	0.170	−21.305
ETo/CSm	2.35 × 10^−6^	1.31 × 10^−5^	−0.002
REo/CSm	3.82 × 10^−5^	−3.41 × 10^−5^	0
PIabs	0.028	−0.003	2.915
PItotal	−0.018	0.001	−1.993
SPAD	0.007	0.001	0.079

**Table 7:** Comparison of test materials.

Genotype	Variety Name	Combination	Average Fertility Period (Day)	Varietal Origin
W01	L9779	—	102.5	Inner Mongolia Autonomous Region, China
W02	KC4	Kefeng No. 6/Ke 87-266	104.5	Heilongjiang Province, China
W03	YL4	Sonora 64/Grand Design	100.5	Ningxia Hui Autonomous Region, China
W04	NM2	Ning 1608/Mengjian 3	100.5	Inner Mongolia Autonomous Region, China
W05	12W145	Yong 754/Guan 35	94.5	Inner Mongolia Autonomous Region, China
W06	GC007	—	96.5	Veracruz, Mexico

Note: — Indicates that parental information is unclear.

**Table 8:** Composition and properties of various salt solutions.

Salt Type	Concentration (mmol·L^−1^)	Composition of Salt and Molar Ratios	pH Value
Neutral salt stress	0	NaCl:Na_2_SO_4_ = 1:1	5.67
	50		5.84
	100		5.95
	150		5.9
	200		6.08
	250		5.88
Alkali salt stress	0	Na_2_CO_3_:NaHCO_3_ = 1:1	5.67
	50		10.26
	100		10.43
	150		10.34
	200		10.32
	250		10.18
Combined salt stress	0	NaCl:Na_2_SO_4_:Na_2_CO_3_:NaHCO_3_ = 1:1:1:1	5.67
	50		10.09
	100		10.2
	150		10.21
	200		10.17
	250		10.20

**Table 9:** Test measurements and their abbreviations.

Classification of Indicators	Abridge	Concrete Meaning
Seedling character	PH	Plant height
	FW	Fresh weight
	DW	Dry weight
	RCR	Root-crown ratio
Gas exchange parameter (GEP)	A	Net photosynthetic rate
	E	Rate of transpiration
	Ci	Intercellular CO_2_ concentration
	GSW	Stomatal conductance
Chlorophyll fluorescence parameters (CFPS)	Fv/Fm	Maximum quantum yield of the primary photochemical reaction at t = 0
	Sm	Receptor bank capacity
	ABS/RC	Light energy absorbed per reaction center unit
	DIo/RC	Total energy released by a single active reaction center
	TRo/RC	Energy captured by the unit’s reaction center for reducing coenzyme A (QA)
	ETo/RC	Energy transferred per reaction center unit
	REo/RC	Flux of electrons transferred from a single active reaction center to an electron acceptor at the end of photosystem I (PS I) for its reduction
	psi (Eo)	The efficiency of single excitons captured by active reaction centers to drive electron transfer, excluding reduced coenzyme A, at the onset of illumination
	phi (Eo)	Quantum efficiency of electron transfer from QA to the electron acceptor in the electron transport chain, excluding QA, at the start of illumination
	delta (Ro)	The efficiency of a single exciton captured by an active reaction center to transfer a single electron from QA through the electron transport chain to the terminal electron acceptor on the PS I receptor side at the start of illumination
	phi (Ro)	Quantum efficiency of PS I receptor-side terminal electron acceptor reduction
	ETo/CSm	At the moment when the fluorescence measurement reaches its maximum (t = t_Fm_), the energy per unit leaf area used for electron transfer
	REo/CSm	Energy flux is transferred from a single active reaction center to the PSI terminal electron acceptor to reduce it at t = t_Fm_.
	PIabs	Performance parameters derived from absorbed light energy
	PItotal	Overall functional activity of PSII, PSI, and the intersystem electron transport chain
Chlorophyll content (Chl C)	SPAD	Relative chlorophyll levels content

## Data Availability

The original contributions presented in this study are included in the article/Supplementary Materials. Further inquiries can be directed to the corresponding author.

## Supplementary Materials

The following supporting information can be downloaded at https://www.mdpi.com/article/10.3390/ijms27073060/s1.

## Abbreviations

The following abbreviations are used in this manuscript:

GEP	Gas exchange parameter
CFPS	Chlorophyll fluorescence parameters
Chl C	Chlorophyll content
BM	Biomass
ST	Salt type
G	Genotype
SC	Salt concentration
STI	Salt tolerance index
SDI	Salt damage index
SEM	Structural equation modeling
NS	Neutral salt stress
AS	Alkali salt stress
CS	Combined salt stress
VIP	Variable importance in the projection
RCs	Regression coefficients
PLSR	Partial least squares regression
RDA	Redundancy analysis

## Appendix A

**Table A1:** FW responses of seedlings to salt concentration gradients under various salt-stress conditions.

Genotypes	Neutral salt concentration (mmol·L^−1^)					
	0	50	100	150	200	250
W01	0.68 ± 0.06 ^a^	0.36 ± 0.02 ^bc^	0.30 ± 0.01 ^a^	0.28 ± 0.02 ^bc^	0.22 ± 0.02 ^ab^	0.21 ± 0.03 ^ab^
W02	0.46 ± 0.06 ^b^	0.26 ± 0.02 ^c^	0.26 ± 0.02 ^a^	0.24 ± 0.01 ^c^	0.16 ± 0.04 ^b^	0.14 ± 0.02 ^b^
W03	0.60 ± 0.05 ^ab^	0.52 ± 0.04 ^ab^	0.42 ± 0.04 ^a^	0.35 ± 0.04 ^ab^	0.27 ± 0.03 ^ab^	0.25 ± 0.03 ^a^
W04	0.62 ± 0.03 ^ab^	0.56 ± 0.07 ^a^	0.37 ± 0.04 ^a^	0.36 ± 0.03 ^ab^	0.32 ± 0.06 ^ab^	0.31 ± 0.04 ^a^
W05	0.63 ± 0.09 ^ab^	0.61 ± 0.09 ^a^	0.45 ± 0.05 ^a^	0.41 ± 0.05 ^a^	0.37 ± 0.06 ^a^	0.26 ± 0.05 ^a^
W06	0.59 ± 0.04 ^ab^	0.44 ± 0.09 ^abc^	0.39 ± 0.12 ^a^	0.35 ± 0.03 ^ab^	0.29 ± 0.09 ^ab^	0.26 ± 0.03 ^a^
Genotypes	Alkaline salt concentration (mmol·L^−1^)					
	0	50	100	150	200	250
W01	0.68 ± 0.06 ^a^	0.25 ± 0.03 ^ab^	0.22 ± 0.02 ^a^	0.22 ± 0.04 ^a^	0.18 ± 0.01 ^a^	0.16 ± 0.02 ^ab^
W02	0.46 ± 0.06 ^b^	0.19 ± 0.03 ^b^	0.18 ± 0.02 ^a^	0.15 ± 0.03 ^a^	0.14 ± 0.01 ^a^	0.12 ± 0.01 ^b^
W03	0.60 ± 0.05 ^ab^	0.26 ± 0.03 ^ab^	0.23 ± 0.02 ^a^	0.21 ± 0.04 ^a^	0.19 ± 0.02 ^a^	0.18 ± 0.01 ^a^
W04	0.62 ± 0.03 ^ab^	0.26 ± 0.04 ^ab^	0.25 ± 0.03 ^a^	0.21 ± 0.04 ^a^	0.19 ± 0.04 ^a^	0.16 ± 0.02 ^ab^
W05	0.63 ± 0.09 ^ab^	0.31 ± 0.04 ^a^	0.20 ± 0.01 ^a^	0.19 ± 0.05 ^a^	0.15 ± 0.03 ^a^	0.15 ± 0.01 ^ab^
W06	0.59 ± 0.04 ^ab^	0.24 ± 0.02 ^ab^	0.21 ± 0.02 ^a^	0.20 ± 0.04 ^a^	0.19 ± 0.02 ^a^	0.18 ± 0.03 ^a^
Genotypes	Combined salt concentration (mmol·L^−1^)					
	0	50	100	150	200	250
W01	0.68 ± 0.06 ^a^	0.33 ± 0.05 ^abc^	0.23 ± 0.03 ^abc^	0.22 ± 0.02 ^abc^	0.22 ± 0.00 ^b^	0.21 ± 0.02 ^ab^
W02	0.46 ± 0.06 ^b^	0.21 ± 0.02 ^c^	0.18 ± 0.01 ^c^	0.18 ± 0.02 ^c^	0.17 ± 0.01 ^c^	0.14 ± 0.01 ^b^
W03	0.60 ± 0.05 ^ab^	0.28 ± 0.04 ^bc^	0.21 ± 0.05 ^bc^	0.20 ± 0.02 ^bc^	0.19 ± 0.01 ^bc^	0.16 ± 0.03 ^ab^
W04	0.62 ± 0.03 ^ab^	0.41 ± 0.04 ^a^	0.24 ± 0.03 ^abc^	0.23 ± 0.05 ^abc^	0.23 ± 0.01 ^b^	0.21 ± 0.01 ^ab^
W05	0.63 ± 0.09 ^ab^	0.39 ± 0.03 ^ab^	0.32 ± 0.01 ^a^	0.31 ± 0.02 ^a^	0.30 ± 0.03 ^a^	0.25 ± 0.04 ^a^
W06	0.59 ± 0.04 ^ab^	0.33 ± 0.05 ^abc^	0.30 ± 0.03 ^ab^	0.29 ± 0.03 ^ab^	0.24 ± 0.01 ^b^	0.22 ± 0.03 ^ab^

Note: Different letters (a, b, c) indicate significant differences at *p* < 0.05 (Duncan’s multiple range test), while the same letter indicates no significant difference. The letters are assigned in descending order of the mean values.

**Table A2:** DW responses of seedlings to salt concentration gradients under various salt-stress conditions.

Genotypes	Neutral salt concentration (mmol·L^−1^)					
	0	50	100	150	200	250
W01	0.06 ± 0.00 ^a^	0.04 ± 0.00 ^ab^	0.04 ± 0.00 ^ab^	0.04 ± 0.00 ^a^	0.03 ± 0.00 ^ab^	0.03 ± 0.00 ^a^
W02	0.04 ± 0.00 ^a^	0.04 ± 0.00 ^b^	0.04 ± 0.00 ^b^	0.03 ± 0.00 ^a^	0.02 ± 0.00 ^b^	0.02 ± 0.00 ^a^
W03	0.05 ± 0.00 ^a^	0.05 ± 0.00 ^ab^	0.04 ± 0.01 ^ab^	0.04 ± 0.00 ^a^	0.04 ± 0.00 ^ab^	0.03 ± 0.01 ^a^
W04	0.05 ± 0.00 ^a^	0.05 ± 0.00 ^a^	0.04 ± 0.00 ^ab^	0.04 ± 0.00 ^a^	0.04 ± 0.00 ^ab^	0.03 ± 0.01 ^a^
W05	0.06 ± 0.00 ^a^	0.06 ± 0.01 ^a^	0.05 ± 0.00 ^a^	0.04 ± 0.01 ^a^	0.04 ± 0.00 ^a^	0.04 ± 0.00 ^a^
W06	0.05 ± 0.01 ^a^	0.05 ± 0.01 ^ab^	0.04 ± 0.00 ^ab^	0.05 ± 0.01 ^a^	0.04 ± 0.01 ^a^	0.03 ± 0.00 ^a^
Genotypes	Alkaline salt concentration (mmol·L^−1^)					
	0	50	100	150	200	250
W01	0.06 ± 0.00 ^a^	0.05 ± 0.00 ^a^	0.05 ± 0.00 ^a^	0.04 ± 0.01 ^a^	0.03 ± 0.00 ^a^	0.03 ± 0.00 ^bc^
W02	0.04 ± 0.00 ^a^	0.04 ± 0.01 ^a^	0.04 ± 0.00 ^a^	0.03 ± 0.01 ^a^	0.03 ± 0.00 ^a^	0.03 ± 0.00 ^c^
W03	0.05 ± 0.00 ^a^	0.05 ± 0.00 ^a^	0.05 ± 0.00 ^a^	0.04 ± 0.01 ^a^	0.04 ± 0.00 ^a^	0.04 ± 0.00 ^ab^
W04	0.05 ± 0.00 ^a^	0.05 ± 0.00 ^a^	0.05 ± 0.00 ^a^	0.05 ± 0.01 ^a^	0.04 ± 0.01 ^a^	0.03 ± 0.00 ^bc^
W05	0.06 ± 0.00 ^a^	0.06 ± 0.01 ^a^	0.05 ± 0.00 ^a^	0.04 ± 0.01 ^a^	0.04 ± 0.00 ^a^	0.04 ± 0.00 ^a^
W06	0.05 ± 0.01 ^a^	0.05 ± 0.00 ^a^	0.05 ± 0.01 ^a^	0.05 ± 0.01 ^a^	0.04 ± 0.01 ^a^	0.04 ± 0.00 ^ab^
Genotypes	Combined salt concentration (mmol·L^−1^)					
	0	50	100	150	200	250
W01	0.06 ± 0.00 ^a^	0.06 ± 0.00 ^a^	0.05 ± 0.00 ^a^	0.05 ± 0.00 ^a^	0.04 ± 0.01 ^a^	0.04 ± 0.01 ^a^
W02	0.04 ± 0.00 ^a^	0.04 ± 0.01 ^a^	0.04 ± 0.00 ^a^	0.04 ± 0.00 ^a^	0.03 ± 0.00 ^a^	0.03 ± 0.00 ^a^
W03	0.05 ± 0.00 ^a^	0.05 ± 0.00 ^a^	0.05 ± 0.00 ^a^	0.04 ± 0.00 ^a^	0.04 ± 0.01 ^a^	0.04 ± 0.00 ^a^
W04	0.05 ± 0.00 ^a^	0.06 ± 0.00 ^a^	0.05 ± 0.01 ^a^	0.05 ± 0.00 ^a^	0.04 ± 0.00 ^a^	0.05 ± 0.00 ^a^
W05	0.06 ± 0.00 ^a^	0.06 ± 0.01 ^a^	0.05 ± 0.01 ^a^	0.05 ± 0.00 ^a^	0.05 ± 0.00 ^a^	0.04 ± 0.00 ^a^
W06	0.05 ± 0.01 ^a^	0.06 ± 0.01 ^a^	0.05 ± 0.00 ^a^	0.05 ± 0.00 ^a^	0.05 ± 0.01 ^a^	0.04 ± 0.01 ^a^

Note: Different letters (a, b, c) indicate significant differences at *p* < 0.05 (Duncan’s multiple range test), while the same letter indicates no significant difference. The letters are assigned in descending order of the mean values.

**Table A3:** SPAD responses of seedlings to salt concentration gradients under various salt-stress conditions.

Genotypes	Neutral salt concentration (mmol·L^−1^)					
	0	50	100	150	200	250
W01	35.40 ± 2.79 ^a^	31.75 ± 2.61 ^a^	29.68 ± 1.74 ^a^	29.23 ± 1.31 ^a^	23.85 ± 3.75 ^a^	20.45 ± 5.21 ^a^
W02	34.00 ± 1.72 ^a^	31.10 ± 2.72 ^a^	29.80 ± 1.97 ^a^	29.58 ± 1.05 ^a^	17.18 ± 3.28 ^a^	16.58 ± 4.14 ^a^
W03	34.33 ± 0.56 ^a^	33.18 ± 0.69 ^a^	31.55 ± 1.60 ^a^	31.00 ± 1.49 ^a^	20.20 ± 2.01 ^a^	17.48 ± 4.17 ^a^
W04	33.83 ± 1.18 ^a^	31.60 ± 0.94 ^a^	27.53 ± 1.16 ^a^	26.23 ± 1.60 ^a^	25.10 ± 3.80 ^a^	18.80 ± 0.88 ^a^
W05	36.40 ± 0.73 ^a^	33.60 ± 2.42 ^a^	32.23 ± 0.25 ^a^	27.10 ± 1.35 ^a^	24.80 ± 2.94 ^a^	24.03 ± 1.29 ^a^
W06	33.20 ± 1.59 ^a^	30.18 ± 2.29 ^a^	28.78 ± 3.26 ^a^	28.20 ± 3.36 ^a^	23.68 ± 1.07 ^a^	22.63 ± 2.30 ^a^
Genotypes	Alkaline salt concentration (mmol·L^−1^)					
	0	50	100	150	200	250
W01	35.40 ± 2.79 ^a^	21.33 ± 4.03 ^a^	11.55 ± 1.68 ^ab^	6.73 ± 2.72 ^a^	3.65 ± 1.69 ^a^	2.83 ± 0.97 ^a^
W02	34.00 ± 1.72 ^a^	9.80 ± 2.80 ^b^	4.48 ± 1.89 ^c^	4.35 ± 2.10 ^a^	3.75 ± 1.18 ^a^	2.25 ± 1.82 ^a^
W03	34.33 ± 0.56 ^a^	14.75 ± 1.90 ^ab^	13.45 ± 1.23 ^a^	5.60 ± 1.20 ^a^	5.48 ± 0.51 ^a^	2.18 ± 0.74 ^a^
W04	33.83 ± 1.18 ^a^	11.75 ± 2.13 ^b^	11.20 ± 2.72 ^ab^	8.18 ± 2.03 ^a^	5.65 ± 2.45 ^a^	3.80 ± 2.00 ^a^
W05	36.40 ± 0.73 ^a^	10.43 ± 1.03 ^b^	6.98 ± 1.27 ^bc^	5.43 ± 3.10 ^a^	4.25 ± 2.04 ^a^	1.88 ± 1.24 ^a^
W06	33.20 ± 1.59 ^a^	13.45 ± 1.20 ^b^	9.90 ± 2.29 ^abc^	8.85 ± 2.20 ^a^	6.78 ± 2.24 ^a^	4.73 ± 0.68 ^a^
Genotypes	Combined salt concentration (mmol·L^−1^)					
	0	50	100	150	200	250
W01	35.40 ± 2.79 ^a^	22.35 ± 1.74 ^a^	9.45 ± 1.58 ^ab^	5.43 ± 0.95 ^b^	5.03 ± 2.15 ^ab^	4.10 ± 1.52 ^b^
W02	34.00 ± 1.72 ^a^	12.58 ± 0.76 ^b^	6.28 ± 3.13 ^b^	2.75 ± 0.58 ^b^	2.65 ± 0.88 ^b^	1.05 ± 0.15 ^b^
W03	34.33 ± 0.56 ^a^	19.18 ± 2.84 ^ab^	8.20 ± 2.65 ^ab^	6.90 ± 1.28 ^b^	5.23 ± 1.36 ^ab^	2.53 ± 0.79 ^b^
W04	33.83 ± 1.18 ^a^	19.08 ± 2.46 ^ab^	6.30 ± 0.53 ^b^	5.00 ± 1.37 ^b^	4.78 ± 0.54 ^ab^	1.70 ± 0.46 ^b^
W05	36.40 ± 0.73 ^a^	20.08 ± 0.61 ^a^	12.70 ± 2.99 ^ab^	8.33 ± 2.59 ^ab^	5.00 ± 1.65 ^ab^	3.25 ± 0.92 ^b^
W06	33.20 ± 1.59 ^a^	21.35 ± 3.73 ^a^	16.40 ± 3.39 ^a^	13.93 ± 3.91 ^a^	8.88 ± 2.59 ^a^	8.48 ± 2.07 ^a^

Note: Different letters (a, b, c) indicate significant differences at *p* < 0.05 (Duncan’s multiple range test), while the same letter indicates no significant difference. The letters are assigned in descending order of the mean values.

**Table A4:** A responses of seedlings to salt concentration gradients under various salt-stress conditions.

Genotypes	Neutral salt concentration (mmol·L^−1^)					
	0	50	100	150	200	250
W01	26.10 ± 0.53 ^a^	11.61 ± 2.09 ^a^	7.72 ± 0.96 ^a^	7.03 ± 2.18 ^a^	6.10 ± 1.28 ^a^	0.38 ± 0.89 ^a^
W02	15.99 ± 0.55 ^c^	15.30 ± 2.21 ^a^	9.92 ± 1.74 ^a^	3.95 ± 0.95 ^a^	6.45 ± 0.92 ^a^	9.45 ± 3.79 ^a^
W03	13.97 ± 0.90 ^cd^	10.06 ± 1.13 ^a^	6.82 ± 1.07 ^a^	4.22 ± 1.02 ^a^	1.66 ± 0.49 ^a^	1.48 ± 3.25 ^a^
W04	12.08 ± 0.31 ^d^	13.04 ± 0.21 ^a^	6.63 ± 0.25 ^a^	5.08 ± 1.39 ^a^	4.86 ± 1.06 ^a^	3.84 ± 2.13 ^a^
W05	24.17 ± 0.89 ^a^	13.28 ± 0.54 ^a^	7.11 ± 0.36 ^a^	5.47 ± 1.63 ^a^	3.40 ± 1.01 ^a^	1.21 ± 0.19 ^a^
W06	19.55 ± 0.37 ^b^	13.05 ± 0.77 ^a^	5.78 ± 1.05 ^a^	1.69 ± 0.44 ^a^	1.70 ± 1.03 ^a^	1.16 ± 0.76 ^a^
Genotypes	Alkaline salt concentration (mmol·L^−1^)					
	0	50	100	150	200	250
W01	26.10 ± 0.53 ^a^	6.65 ± 0.75 ^a^	3.07 ± 0.36 ^abc^	1.54 ± 0.93 ^a^	-	-
W02	15.99 ± 0.55 ^c^	4.97 ± 0.38 ^ab^	4.34 ± 0.51 ^ab^	0.03 ± 1.79 ^a^	0.59 ± 2.01 ^a^	-
W03	13.97 ± 0.90 ^cd^	5.83 ± 0.12 ^a^	1.30 ± 0.77 ^bc^	0.58 ± 1.10 ^a^	-	-
W04	12.08 ± 0.31 ^d^	6.18 ± 0.20 ^a^	4.34 ± 0.45 ^ab^	0.52 ± 0.34 ^a^	-	-
W05	24.17 ± 0.89 ^a^	6.73 ± 0.45 ^a^	5.15 ± 0.79 ^a^	0.26 ± 0.34 ^a^	-	-
W06	19.55 ± 0.37 ^b^	0.99 ± 1.63 ^b^	0.14 ± 0.48 ^c^	-	-	-
Genotypes	Combined salt concentration (mmol·L^−1^)					
	0	50	100	150	200	250
W01	26.10 ± 0.53 ^a^	7.05 ± 5.95 ^a^	2.19 ± 4.48 ^a^	2.06 ± 1.02 ^a^	1.75 ± 0.21 ^a^	1.55 ± 0.41 ^a^
W02	15.99 ± 0.55 ^c^	1.52 ± 9.62 ^a^	1.04 ± 3.45 ^a^	0.12 ± 1.89 ^a^	0.05 ± 0.19 ^a^	-
W03	13.97 ± 0.90 ^cd^	5.79 ± 1.17 ^a^	0.26 ± 0.55 ^a^	-	-	-
W04	12.08 ± 0.31 ^d^	4.25 ± 3.16 ^a^	1.42 ± 5.47 ^a^	0.04 ± 2.13 ^a^	-	-
W05	24.17 ± 0.89 ^a^	8.18 ± 1.53 ^a^	1.19 ± 1.73 ^a^	0.54 ± 3.57 ^a^	0.39 ± 2.36 ^a^	-
W06	19.55 ± 0.37b	9.02 ± 2.12a	3.62 ± 1.51a	2.94 ± 0.81a	-	-

Note: - indicates values too small to be statistically significant.

**Table A5:** Fv/Fm responses of seedlings to salt concentration gradients under various salt-stress conditions.

Genotypes	Neutral salt concentration (mmol·L^−1^)					
	0	50	100	150	200	250
W01	0.81 ± 0.01 ^a^	0.82 ± 0.00 ^a^	0.82 ± 0.00 ^a^	0.83 ± 0.00 ^a^	0.72 ± 0.07 ^a^	0.41 ± 0.23 ^a^
W02	0.78 ± 0.02 ^a^	0.80 ± 0.00 ^b^	0.81 ± 0.00 ^b^	0.81 ± 0.01 ^a^	0.03 ± 0.00 ^b^	0.58 ± 0.19 ^a^
W03	0.81 ± 0.00 ^a^	0.81 ± 0.00 ^a^	0.81 ± 0.00 ^ab^	0.80 ± 0.00 ^a^	0.58 ± 0.19 ^a^	0.58 ± 0.13 ^a^
W04	0.81 ± 0.00 ^a^	0.82 ± 0.00 ^a^	0.80 ± 0.00 ^b^	0.82 ± 0.00 ^a^	0.81 ± 0.00 ^a^	0.80 ± 0.00 ^a^
W05	0.80 ± 0.00 ^a^	0.82 ± 0.00 ^a^	0.82 ± 0.00 ^ab^	0.53 ± 0.17 ^b^	0.80 ± 0.01 ^a^	0.82 ± 0.00 ^a^
W06	0.81 ± 0.01 ^a^	0.83 ± 0.00 ^a^	0.82 ± 0.00 ^ab^	0.80 ± 0.01 ^a^	0.63 ± 0.10 ^a^	0.81 ± 0.00 ^a^
Genotypes	Alkaline salt concentration (mmol·L^−1^)					
	0	50	100	150	200	250
W01	0.81 ± 0.01 ^a^	0.72 ± 0.05 ^a^	0.01 ± 0.00 ^b^	0.02 ± 0.00 ^b^	0.02 ± 0.00 ^a^	0.02 ± 0.00 ^ab^
W02	0.78 ± 0.02 ^a^	0.21 ± 0.18 ^b^	0.03 ± 0.00 ^b^	0.01 ± 0.00 ^c^	0.02 ± 0.00 ^a^	0.03 ± 0.00 ^a^
W03	0.81 ± 0.00 ^a^	0.25 ± 0.10 ^b^	0.21 ± 0.18 ^b^	0.01 ± 0.00 ^bc^	0.02 ± 0.00 ^a^	0.03 ± 0.01 ^a^
W04	0.81 ± 0.00 ^a^	0.77 ± 0.01 ^a^	0.13 ± 0.02 ^b^	0.01 ± 0.00 ^c^	0.02 ± 0.00 ^a^	0.03 ± 0.00 ^a^
W05	0.80 ± 0.00 ^a^	0.14 ± 0.09 ^b^	0.01 ± 0.00 ^b^	0.02 ± 0.00 ^b^	0.02 ± 0.00 ^a^	0.01 ± 0.00 ^b^
W06	0.81 ± 0.01 ^a^	0.72 ± 0.05 ^a^	0.56 ± 0.18 ^a^	0.03 ± 0.00 ^a^	0.02 ± 0.00 ^a^	0.02 ± 0.00 ^ab^
Genotypes	Combined salt concentration (mmol·L^−1^)					
	0	50	100	150	200	250
W01	0.81 ± 0.01 ^a^	0.66 ± 0.11 ^b^	0.02 ± 0.00 ^c^	0.33 ± 0.18 ^ab^	0.07 ± 0.04 ^a^	0.01 ± 0.00 ^a^
W02	0.78 ± 0.02 ^a^	0.78 ± 0.00 ^ab^	0.04 ± 0.00 ^c^	0.02 ± 0.00 ^b^	0.11 ± 0.09 ^a^	0.22 ± 0.19 ^a^
W03	0.81 ± 0.00 ^a^	0.81 ± 0.01 ^ab^	0.72 ± 0.00 ^a^	0.01 ± 0.00 ^b^	0.02 ± 0.01 ^a^	0.02 ± 0.00 ^a^
W04	0.81 ± 0.00 ^a^	0.80 ± 0.01 ^ab^	0.31 ± 0.15 ^b^	0.14 ± 0.13 ^b^	0.01 ± 0.00 ^a^	0.02 ± 0.00 ^a^
W05	0.80 ± 0.00 ^a^	0.81 ± 0.01 ^a^	0.05 ± 0.01 ^c^	0.01 ± 0.00 ^b^	0.01 ± 0.00 ^a^	0.01 ± 0.00 ^a^
W06	0.81 ± 0.01 ^a^	0.80 ± 0.00 ^ab^	0.15 ± 0.08 ^bc^	0.51 ± 0.16 ^a^	0.11 ± 0.04 ^a^	0.01 ± 0.00 ^a^

Note: Different letters (a, b, c) indicate significant differences at *p* < 0.05 (Duncan’s multiple range test), while the same letter indicates no significant difference. The letters are assigned in descending order of the mean values.

**Table A6:** Genotype-specific linear regression equations and salt-tolerance thresholds for biomass STI (FW, PH) across salt-stress gradients [0–250 mM].

Genotypes	PH Regression Equation	R^2^	*p*-Value	Salt Tolerance Threshold (mmol·L^−1^)	Genotypes	FW Regression Equation	R^2^	*p*-Value	Salt Tolerance Threshold (mmol·L^−1^)
Neutral salt stress									
W01	Y = 0.93 − 0.002X	0.88	***p* < 0.01**	215	W01	Y = 0.79 − 0.002X	0.72	***p* < 0.05**	145
W02	Y = 0.91 − 0.001X	0.85	***p* < 0.01**	410	W02	Y = 0.86 − 0.002X	0.83	***p* < 0.05**	180
W03	Y = 0.99 − 0.002X	0.99	***p* < 0.01**	245	W03	Y = 0.97 − 0.002X	0.98	***p* < 0.01**	235
W04	Y = 0.98 − 0.002X	0.95	***p* < 0.01**	240	W04	Y = 0.95 − 0.002X	0.85	***p* < 0.01**	225
W05	Y = 0.91 − 0.001X	0.91	***p* < 0.01**	220	W05	Y = 1.02 − 0.002X	0.96	***p* < 0.01**	260
W06	Y = 0.97 − 0.002X	0.97	***p* < 0.01**	235	W06	Y = 0.91 − 0.002X	0.92	***p* < 0.01**	205
Total mean salt tolerance threshold (mmol·L^−1^)					235				
Alkaline salt stress									
W01	Y = 0.84 − 0.002X	0.74	***p* < 0.05**	170	W01	Y = 0.72 − 0.002X	0.59	0.07	110
W02	Y = 0.85 − 0.002X	0.67	***p* < 0.05**	175	W02	Y = 0.74 − 0.002X	0.64	0.06	120
W03	Y = 0.91 − 0.002X	0.88	***p* < 0.01**	205	W03	Y = 0.74 − 0.002X	0.61	0.07	120
W04	Y = 0.84 − 0.002X	0.72	***p* < 0.05**	170	W04	Y = 0.74 − 0.002X	0.63	0.06	120
W05	Y = 0.82 − 0.002X	0.62	0.06	160	W05	Y = 0.76 − 0.003X	0.70	***p* < 0.05**	87
W06	Y = 0.84 − 0.002X	0.73	***p* < 0.05**	170	W06	Y = 0.72 − 0.002X	0.54	0.09	110
Total mean salt tolerance threshold (mmol·L^−1^)					143				
Combined salt stress									
W01	Y = 0.84 − 0.002X	0.66	***p* < 0.05**	170	W01	Y = 0.74 − 0.002X	0.60	0.07	120
W02	Y = 0.89 − 0.002X	0.78	***p* < 0.05**	195	W02	Y = 0.75 − 0.002X	0.60	0.07	125
W03	Y = 1.03 − 0.002X	0.95	***p* < 0.01**	265	W03	Y = 0.75 − 0.002X	0.64	0.06	125
W04	Y = 0.91 − 0.002X	0.87	***p* < 0.01**	205	W04	Y = 0.83 − 0.002X	0.74	***p* < 0.05**	165
W05	Y = 0.85 − 0.002X	0.71	***p* < 0.05**	175	W05	Y = 0.83 − 0.002X	0.72	***p* < 0.05**	165
W06	Y = 0.87 − 0.002X	0.77	***p* < 0.05**	185	W06	Y = 0.82 − 0.002X	0.73	***p* < 0.05**	160
Total mean salt tolerance threshold (mmol·L^−1^)					171				

Note: Different letters (a, b, c) indicate significant differences at *p* < 0.05 (Duncan’s multiple range test), while the same letter indicates no significant difference. The letters are assigned in descending order of the mean values.

**Table A7:** Partial least squares structural equation modeling (PLS-SEM) hypothesis testing and goodness-of-fit report.

Model Fitting and Predictive Capability Metrics	Control	Neutral Salt	Alkaline Salt	Combined Salt
Standardized Root Mean Square Residual (SRMR)	0.227	0.092	0.089	0.081
Norm Fitting Index (NFI)	0.169	0.630	0.610	0.648
Cross-Validation Redundancy Value (Q^2^_(BM)_)	0.043	0.693	0.654	0.547
